# From sensation to regulation: the diverse functions of peripheral sensory nervous system

**DOI:** 10.3389/fimmu.2025.1575917

**Published:** 2025-05-16

**Authors:** Yixiao Mei, Bing-Lin Zhou, Da Zhong, Xuan-Jie Zheng, Yu-Tao Deng, Lina Yu, Bao-Chun Jiang

**Affiliations:** ^1^ Department of Anesthesiology, The Second Affiliated Hospital, Zhejiang University School of Medicine, Hangzhou, China; ^2^ Zhejiang Key Laboratory of Pain Perception and Neuromodulation, Hangzhou, China; ^3^ State Key Laboratory of Transvascular Implantation Devices, the Second Affiliated Hospital, Zhejiang University School of Medicine, Hangzhou, China

**Keywords:** peripheral sensory nervous system (PNS), sensory signal transduction, nonsensory regulatory functions, neuro-immune interaction, CGRP signaling pathway, microbe-nerve-cell crosstalk

## Abstract

The peripheral sensory nervous system (PNS) has been widely recognized for its role in the collection, processing, and transmission of sensory information, including thermal, mechanical, chemical, and proprioceptive stimuli. In recent years, there has been a growing scholarly interest in the PNS attributable to its multiple physiological and pathophysiological non-sensory roles in the organs it innervates. The PNS exerts regulatory functions within the organs it innervates through direct interactions with local cells or through microbe-nerve-cell interactions that differ from the traditional feedback regulatory modes used by the hormonal and sensory brain-sympathetic/parasympathetic systems. The release of the neuropeptide calcitonin gene related peptide (CGRP) by nerves, through its action on CGRP receptors in peripheral cells, constitutes a primary molecular axis for PNS regulation of organ cells, maintaining tissue homeostasis, facilitating pathological processes, and modulating innate and adaptive immunity. This review highlights the non-sensory functions of the peripheral sensory nervous system in various tissues and organs, focusing on phenotypes, molecular mechanisms and their significance, while also exploring future research directions, methodologies and potential preclinical studies aimed at targeting these pathways for the development of novel therapies.

## Introduction

**Figure 1:**
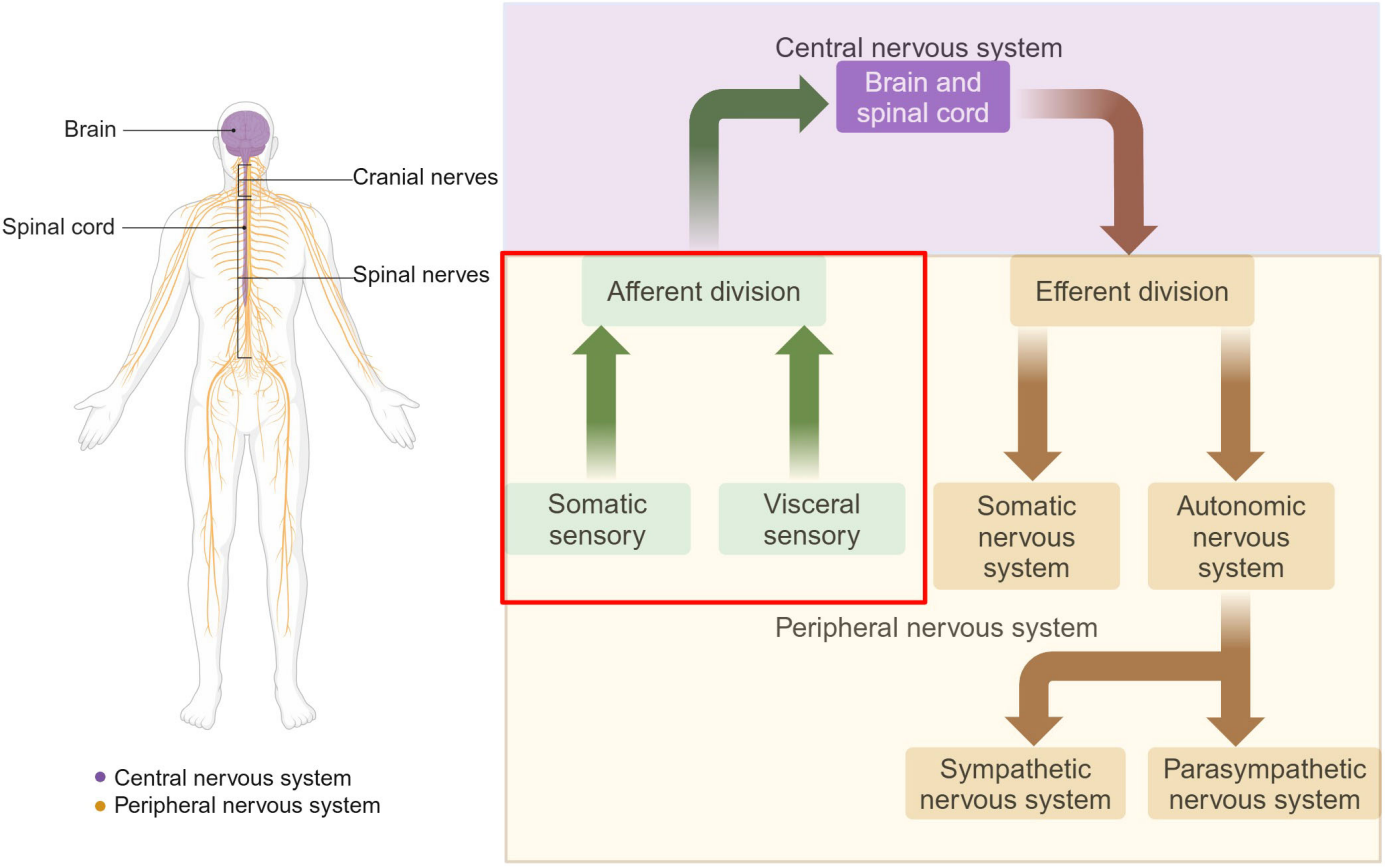
Organization of the nervous system. The nervous system is divided into two main parts: the CNS and the PNS. The CNS is composed of the brain and the spinal cord. The PNS consists of all the neural tissue outside the CNS, including nerves and ganglia. Functionally, the PNS is divided into the somatic and autonomic nervous systems. The sensory nervous system, also known as the afferent nervous system, is a crucial component of the PNS. Somatic sensory includes sensations of touch, pain, pressure, vibration and the special sensations of hearing, equilibrium and vision. Visceral sensory includes stretch, pain, temperature, chemical changes, irritation in viscera, nausea and hunger, as well as special sensations of taste and smell. Adapted from “The Major Components of the Nervous System”, by BioRender.com (2025) Retrieved from https://app.biorender.com/biorender-templates.

The sensory nervous system is a critical component of the PNS, responsible for processing and transmitting information about changes in both external environment and internal organ functions to the central nervous system (CNS). It comprises two major subdivisions: the somatosensory nervous system, which is responsible for detecting and responding to external stimuli such as touch, temperature, chemicals, light and odor (1), and the visceral sensory system, which monitors internal physiological states, including organ function, blood pressure, and internal temperature (2, 3) (**Figure 1**). Therefore, the primary functions of the peripheral sensory nervous system include the transmission of sensory information from the periphery to the CNS by electrical and chemical signals, allowing the body to perceive and respond to changes in both the external and internal environments. This capability is essential for maintaining homeostasis and ensuring survival.

Beyond its well-established role in sensory perception, emerging evidence suggests that the peripheral sensory system can be directly activated by “danger signals”, such as microbial invasion, tissue damage, or harmful environmental stimuli, without necessarily forming conscious sensations (4, 5). These danger signals are distinct from traditional stimuli, like touch or temperature, which typically result in sensory perception and conscious awareness. Instead, danger signals serve as critical indicators of potential threats, triggering immediate physiological responses aimed at protecting the body. Mechanistically, the peripheral sensory neurons, which are extensively innervated within the skin and visceral organs, possess peripheral terminals that are rich in specialized transducer proteins, including transient receptor potential (TRP) channels, G protein-coupled receptors (GPCRs) and pattern recognition receptors (PRRs), such as Toll-like receptors (TLRs) (6–8). These molecular components enable sensory neurons to directly detect and respond to danger signals by initiating protective reflexes and immune responses.

Upon activation by harmful stimuli, these somatosensory neurons engage in more than just sensory transmission; they actively participate in local regulatory processes by releasing neuropeptides and chemokines/cytokines from both their central and peripheral terminals. For instance, neuropeptides like CGRP and chemokines such as chemokine ligand 2 (CCL2) are released and can bind to receptors on immune cells or visceral organs (9, 10). This interaction allows the peripheral sensory neurons to exert direct and localized regulatory effects, thereby facilitating communication between the nervous and immune systems and influencing the functions of other organs. The multifunctional molecules expressed on sensory neurons enable the PNS to operate beyond the realm of sensation.

The sensory system not only monitors overall internal and external changes to maintain body homeostasis but also plays a dual role in direct and localized regulation of organ functions, enabling rapid responses to stimuli. On the other hand, the sensory system transmits crucial information to the CNS as afferents, allowing for comprehensive bodily responses to stimuli. It is well understood that the PNS conveys both conscious sensations (e.g., gut distention, cardiac ischemia, temperature, pain) and unconscious visceral sensations (e.g., blood pressure, chemical composition of the blood). However, recent findings reveal that the PNS also transmits information regarding the microenvironmental status of organs, including immune responses within the lungs and the microbial environment of the gut (11–14). This highlights its essential role in maintaining homeostasis and coordinating complex physiological responses.

However, the functions of the sensory system are not always beneficial. In fact, the multifunctional molecules released by the sensory system may exacerbate certain diseases. For example, the neuropeptide CGRP released by peripheral sensory neurons has been implicated in supporting cancer cell growth (15).

In this review, we will explore the recently discovered functions of the sensory system and delve into the cellular and molecular mechanisms underlying the interactions between the sensory system and various organs, with a particular focus on both the physiological and pathological roles of the PNS in immune responses, organ function, tissue repair, and metabolic regulation.

## Anatomy and traditional physiological function of the sensory system

### Somatosensory nervous system

The somatosensory system, a specialized neural network also termed the general body afferent system, mediates the transmission and integration of sensory modalities including tactile perception, nociception, and proprioception. Somatosensory (primary afferent) neurons, characterized by their pseudounipolar morphology, predominantly reside in peripheral sensory ganglia-specifically the trigeminal ganglion (TG) and dorsal root ganglion (DRG). These specialized cells exhibit bifurcated axons that enable simultaneous signal transmission to central synaptic targets and peripheral innervation fields (16).

From a neuroanatomical perspective, the general somatic afferent nerves of the face and forehead originate from neuronal cell bodies in the TG. Their peripheral axons form the trigeminal nerve, which innervates sensory functions of the head and face, while their central axons terminate in the spinal trigeminal nucleus. In contrast, somatic afferent neurons from the caudal part of the head and the rest of the body are located in the DRG of the spinal cord. Their peripheral axons innervate sensory receptors in the skin, muscles, and internal organs, while their central axons terminate in the spinal dorsal horn or medulla, further transmitting sensory information to higher-order centers for processing (17).

Based on nerve fiber diameter, cell body size, and degree of myelination, the classical classification of somatosensory nerve fibers is as follows. C fibers are the smallest-diameter nerve fibers and are unmyelinated. They are responsible for carrying slow, dull, and diffuse pain sensations, as well as transmitting information related to temperature, especially cold sensations. Aδ fibers are myelinated and have a medium-sized diameter. They are involved in the transmission of sharp, acute pain and play a role in detecting rapid temperature changes. Aβ fibers are also myelinated and have a relatively large diameter. They mainly mediate tactile sensations. In this classification, C fibers and Aδ fibers are attributed to nociceptive fibers, primarily transmitting pain and temperature signals, whereas Aβ fibers mainly mediate tactile sensations (18). Additionally, Aα and Aβ proprioceptors are distributed in muscles and joints, encoding the body’s spatial position and movement status to maintain posture control and coordinated movement (19).

In recent years, key molecular markers and activation characteristics of different sensory neuron subtypes have been gradually identified, deepening our understanding of sensory neuron function. Among them, an important subset of sensory neurons are nociceptors, which are characterized by the expression of various nociceptive ion channels, such as voltage-gated sodium channels and transient receptor potential vanilloid 1 (TRPV1) (20–22). Some nociceptors are thinly myelinated (Aδ fibers), which conduct signals relatively quickly, while most are unmyelinated (C fibers), exhibiting slower conduction speeds. These nociceptive neurons constitute the majority of sensory neurons in the peripheral nervous system and play a key role in transmitting acute and chronic pain signals, contributing significantly to various pathological pain conditions.

### Visceral sensory system

Visceral sensory neurons function to monitor dynamic changes in the internal milieu (as opposed to external environmental stimuli) and maintain physiological homeostasis through regulatory effects on effector organs (23).

**Figure 2:**
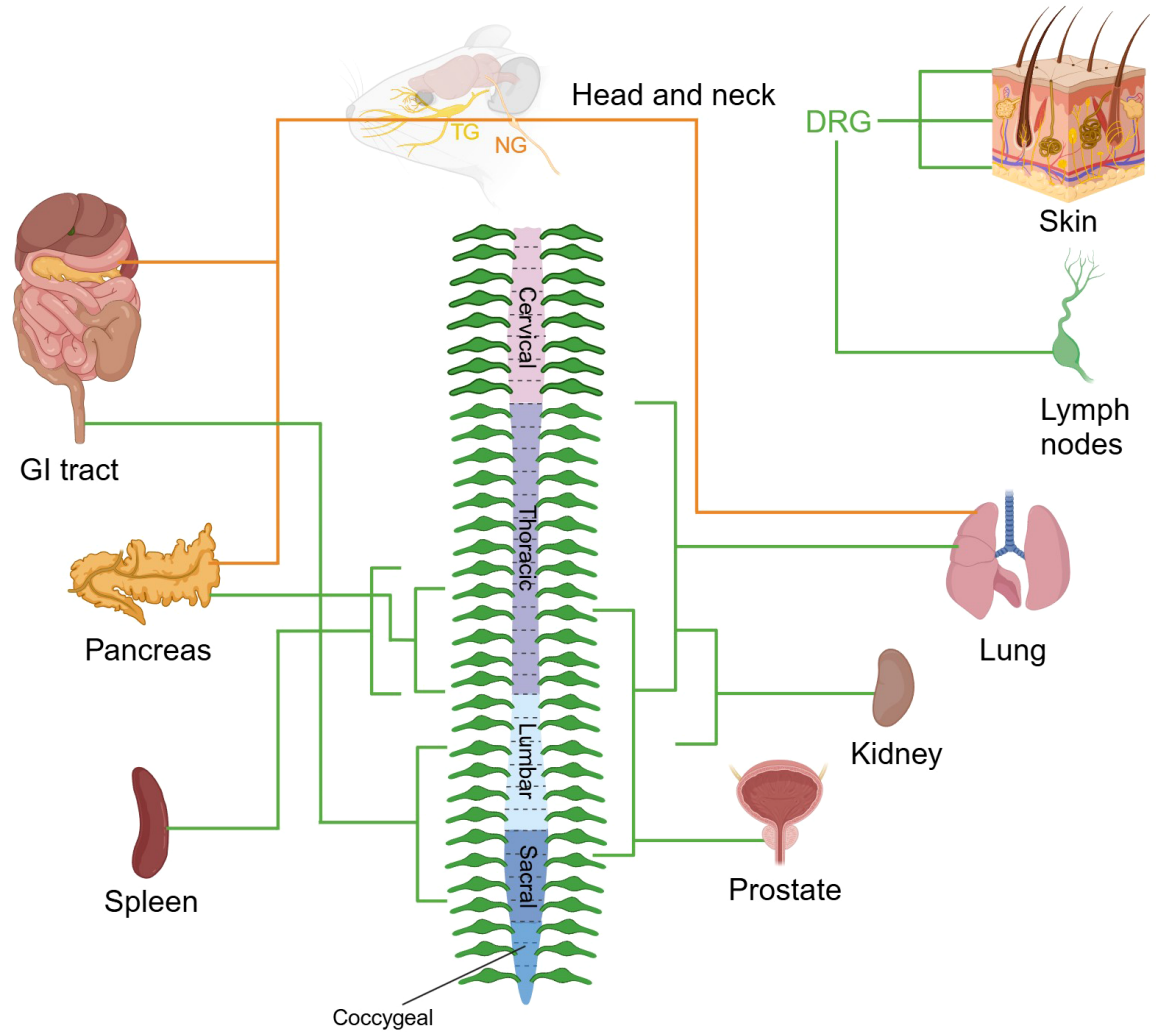
Sensory nerve supply to organs. The images depict the sensory innervation of various mouse organs from DRG, TG, and NG. The peripheral nervous system ganglia are organized symmetrically, and organs often receive innervation from the same ganglia from both sides of the body. Sensory nerve fibers from DRG and NG are marked in green and orange respectively. TG, trigeminal ganglion; NG, nodose ganglion; DRG, dorsal root ganglion; GI, gastrointestinal. Created with BioRender.com.

The somata of this system are bilaterally distributed in ganglia structures, including DRG along with specific cranial nerve ganglia: the geniculate ganglion of the facial nerve (cranial nerves VII), petrosal ganglion of the glossopharyngeal nerve (cranial nerves IX), and nodose ganglion (NG) of the vagus nerve (cranial nerves X). The vagal ganglia (VG), composed of the NG and jugular ganglia (JG), are specialized sensory clusters that differentially process somatosensory signals from the head/neck region (via JG) and viscerosensory inputs from internal organs (via NG) (24). In this section, we focus solely on the innervation of the NG within the viscerosensory system (**Figure 2**).

Anatomically, afferent projections from cranial nerves VII and IX predominantly innervate the tympanic membrane, middle ear cavity, and carotid body, while vagal afferents (cranial nerves X) establish extensive networks in visceral organs including the trachea, pulmonary parenchyma, hepatopancreatic complex, gastrointestinal tract, and bladder. The central projections of visceral afferents from cranial nerves VII, IX, and X predominantly terminate at the nucleus of the solitary tract in the medulla oblongata (25, 26). Visceral sensory fibers originating from DRGs primarily project to lamina V-VII of the spinal dorsal horn, forming multi-level neural networks within corresponding spinal segments.

Murine studies reveal dual sensory innervation patterns in tracheopulmonary, digestive, and pancreatic systems, with neural inputs derived from both segmentally matched DRG neurons and vagal sensory neurons. The pulmonary system receives concurrent innervation from VG and DRGs (27), while gastrointestinal regulation involves coordinated inputs from NG and lumbosacral (L3-S3) DRGs (28). Pancreatic sensory integration arises from NG in combination with thoracic (T9-T13) DRGs (29). Functional analyses demonstrate vagal afferents predominantly mediate mechanoreceptive, chemosensory, and osmosensory transduction underlying reflex responses (e.g., emesis and gastrointestinal motility), whereas DRG neurons encode conscious perception of visceral distension and nociceptive signaling in dually innervated organs (30, 31). Organs with singular innervation patterns exhibit distinct neuroanatomical features: renal nociception transmits via sympathetic pathways to T11-L2 spinal segments (32, 33), while prostatic sensation originates from thoracolumbar (T10-S1) DRGs (34). Recent breakthroughs identified dense nociceptive innervation of the spleen by left-sided T8-T13 DRG-derived fibers (35), and lymph nodes demonstrate specialized innervation by immunomodulatory-capable sensory neuron subpopulations, providing new insights into neuroimmune crosstalk mechanisms (36).

### Similarities and differences between vagal afferent neurons and conventional sensory neurons

Sensory neurons that mediate peripheral regulation originate from distinct ganglia, including the DRG, TG, and vagal ganglia—comprising both NG and JG. Among these, vagal afferents and somatic sensory neurons exhibit both overlapping and divergent anatomical and functional characteristics (37). Vagal afferents and somatic sensory neurons share fundamental structural and signaling features: both are pseudounipolar neurons located in the peripheral ganglia (nodose/jugular ganglia and DRG, respectively), utilize glutamate for neurotransmission, and exhibit neuroplasticity (38, 39). Functionally, vagal afferents innervate visceral organs such as the heart, lungs, and gastrointestinal tract, where they sense mechanical, chemical, and metabolic cues and relay signals to the brainstem (notably the nucleus tractus solitarius) to regulate autonomic and homeostatic processes. In contrast, DRG neurons target somatic tissues including skin and muscle, detect external stimuli such as pain, temperature, and touch, and project to the somatosensory cortex, contributing to conscious perception (40–42).

## Non-sensory functions

### Immune system

Recent studies have revealed that sensory neurons, particularly nociceptors, not only detect harmful stimuli but also actively regulate immune responses (43–48). These neurons innervate key immune organs—such as the bone marrow, spleen, and lymph nodes—and communicate with immune and stromal cells through the release of neuropeptides like CGRP and substance P (22, 49). This interaction is now recognized as forming neuro-immune cell units (NICUs)—anatomical and functional structures where neurons, immune cells, and non-immune cells work together to modulate local immune activity (50–53). Based on this framework, the following sections explore how the PNS engages in organ-specific immune regulation, focusing on its roles within lymphoid and hematopoietic tissues. Neuroimmune interactions in barrier organs such as the skin, lungs, and gut are discussed in later sections. In addition, this section comprehensively summarizes the non-classical functions of sensory neurons in different immune organs and tissues (**Table 1**).

**Table 1:** Interaction between the PNS and the immune systems.

Organs/Tissues	Sensory neuron	Stimulus	Neuropeptides/Neurotransmitters	Interactors	Functions	Ref
Spleen	Nociceptors	PGE2	CGRP	CALCRL-RAMP1 receptor on B cells	Promote humoral immune responses and host defense	(54)
Bone marrow	Nociceptors	/	CGRP	CALCRL-RAMP1 receptor on HSCs	Promote the egress of HSCs	(55)
Lymph nodes	Peptidergic nociceptors	Peripheral Inflammatory	/	Endothelium, stromal cells and innate leukocytes	Monitor LNs and regulate gene expression	(36)
Skin	GINIP neurons	/	TAFA4	IL-10 produced by macrophages	Reduce skin inflammation and promote tissue regeneration	(56)
	NaV1.8 nociceptors	/	CGRP	RAMP1 on neutrophils, monocytes and macrophage	Accelerate wound healing and promote muscle regeneration	(9)
	NaV1.8 nociceptors	Bacterial products	CGRP	Macrophages	Decrease neutrophil and monocyte recruitment	(57)
	TRPV1 nociceptors	Bacterial products	CGRP	Macrophages	Inhibit the recruitment of neutrophils and opsonophagocytic killing of S. pyogenes	(58)
	TRPV1 nociceptors	/	/	A local type 17 immune response	Mediate an anticipatory immune response to prepare the host for pathogen exposure	(59)
	MrgprD neurons	/	Glutamate	Mast cells	Suppress mast cell hyperresponsiveness and skin inflammation	(60)
Lungs	Vagal sensory neurons	Jak1	CGRP	lymphoid cell	Suppresses allergic lung inflammation	(61)
	NaV1.8 nociceptors	IL-5	VIP	CD4^+^ and resident ILC2s	Promotes allergic inflammation	(11)
	Vagal sensory neurons	NMU-Nmurl	NMU	ILC2s	Modulate inflammation	(62)
	TRPV1 nociceptors	Bacterial products	CGRP	Neutrophil and γδ T cell	Suppress pulmonary γδ T cell- and neutrophil-mediated host defense	(63)
Gut	TRPV1 nociceptor	Bacterial (Salmonela)	CGRP	M cell and segmentous filamentous bacteria	Shape the gut microbiota to resist Salmonella infection	(13)
	Sensory neuron	Bacterial products	SP CGRP	Walls of blood vessels	Promotes neurogenic inflammatory responses	(14)
	TRPV1 nociceptor	/	CGRP	RAMP1 receptor on regulatory T cells	Inhibit the proliferation of Tregs in the gut	(64)

Summary the non-classical functions of sensory neurons in different immune organs and tissues.

It includes different neurotransmitters released by sensory neurons after stimulation, as well as interacting substances and biological effects.

#### Neuro-immune cell units

**Figure 3:**
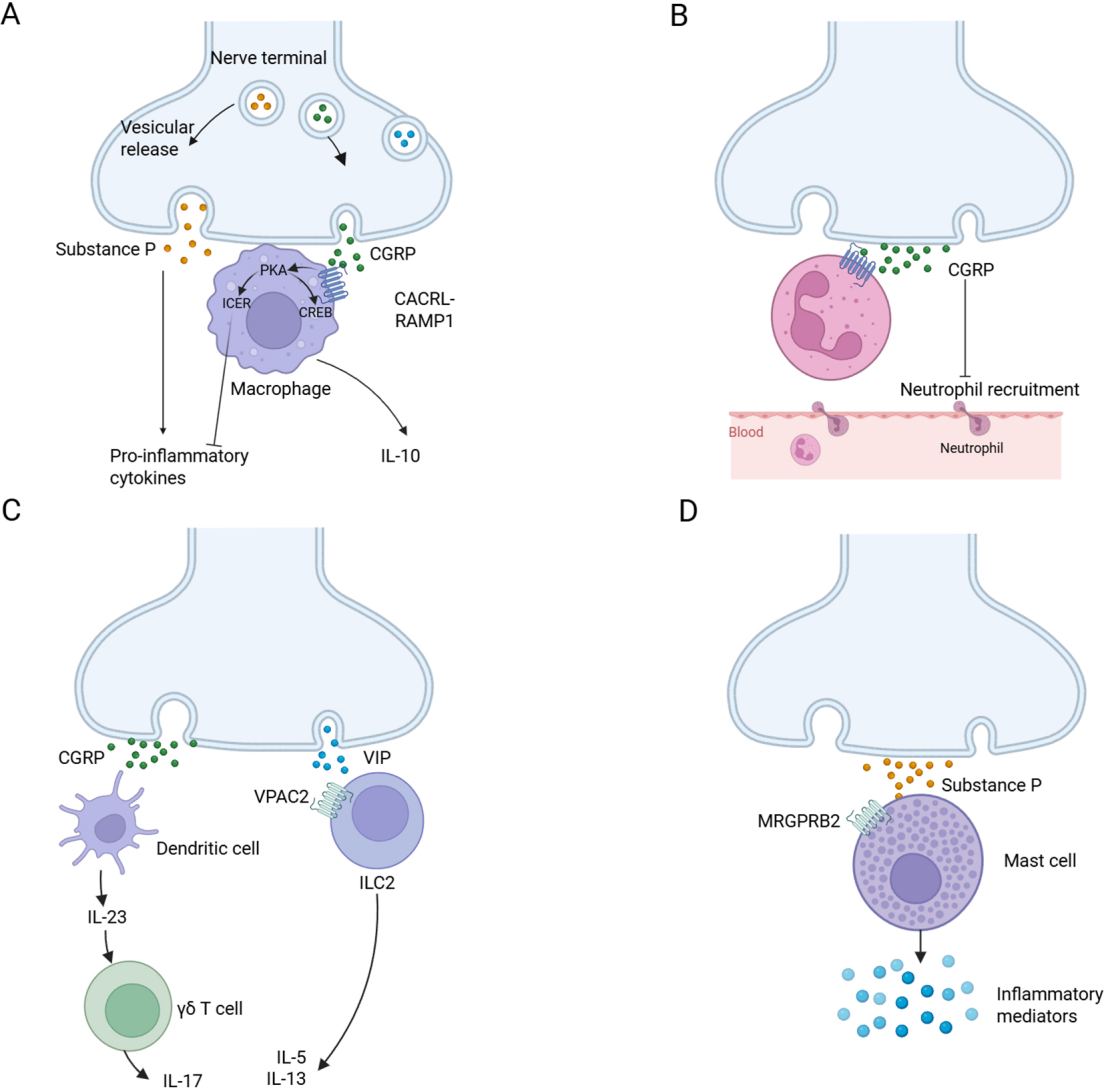
Neuro-immune cell units. **(A)** Substance P is released by nociceptors and acts on monocytes and macrophages through NF-κB activation mediated by ERK-p38 mitogen-activated protein kinase (MAPK), driving the expression of pro-inflammatory cytokines (left). CGRP acts on CALCRL-RAMP1, drives cAMP-protein kinase A (PKA) -dependent CREB regulation, upregulates IL-10 and induces cAMP early repressor (ICER)-dependent transcriptional inhibition of proinflammatory cytokines (right). **(B)** Nociceptors release CGRP, reduce neutrophil recruitment to the lungs and skin, and inhibit the ability of neutrophils to regulate phagocytosis and kill bacteria. **(C)** CGRP from nociceptive neurons drives dermal dendritic cells to produce IL-23, which in turn leads to the activation of γδT cells and the production of IL-17 (left). Neuropeptide vasoactive intestinal peptide (VIP) activates its receptor VPAC2 on ILC2s and drives the production of IL-5 and IL-13 (right). **(D)** Substance P activates MAS-related G protein-coupled receptor member B2 (MrgprB2 receptor) on mast cells, leading to mast cell degranulation and pro-inflammatory mediator release. Created with BioRender.com.

NICUs have been gradually recognized and discovered, which exist in the lymphatic system, adipose tissue, mucosal barrier, and so on. These units are characterized by the co-localization of immune and neuronal cells in defined anatomical locations, allowing for functional interactions that regulate tissue physiology and protection. When harmful or noxious stimuli activate nociceptors, they can regulate the immune response by releasing neurotransmitters (48, 65, 66). Mechanistically, nociceptor activation triggers peripheral neuropeptide secretion (e.g., CGRP, substance P), engaging cognate receptors on myeloid (dendritic cells, macrophages) and lymphoid (T cells) populations, an evolutionarily conserved mechanism forming functional neuroimmune synapses (**Figure 3**). The latest research reveals CGRP-mediated transcriptional reprogramming of dendritic cells, enhancing their sentinel capacity through upregulation of interferon-response genes (e.g., pro-IF-1β) while priming proinflammatory cytokine cascades via calcium-dependent depolarization. Parallel nociceptor-derived CCL2 signaling governs dual-phase immune regulation, synchronizing acute leukocyte recruitment with antigen-presentation efficiency for adaptive immunity induction (67). Other aspects of NICUs have been thoroughly reviewed in Godinho-Silva C's article (65). Ongoing research is uncovering the full extent of NICUs' influence on health and disease. The potential of targeting NICUs for treatment is an exciting frontier in immunology and neuroscience.

#### The neuroimmune interaction is bidirectional

Pathogenic invasion or tissue trauma triggers localized release of inflammatory mediators (e.g., cytokines, nerve growth factor, prostaglandin E2, histamine) from resident and circulating immune populations. Polymodal nociceptive neurons express cognate receptors for these signals at peripheral terminals, with ligand binding initiating dual modulation: 1) neuroplastic changes in both electrical excitability and transcriptional programming; 2) enhanced sensitivity of key nociceptive ion channels (e.g., TRPV1, Nav1.8) through phosphorylation-mediated gating alterations (68). Beyond inflammatory signaling, nociceptors directly detect microbial motifs and cellular breakdown products through pattern recognition receptors, constituting the neurobiological basis of pathogen-induced pain (5). Notably, the innate immune regulator stimulator of interferon genes (STING) is an evolutionarily conserved cytosolic DNA sensor, which orchestrates type I interferon production for antimicrobial and antitumor immunity (69, 70). Emerging research positions this pathway as a hierarchical regulator of nociception, demonstrating its capacity to suppress voltage-gated sodium/calcium channel activity within milliseconds through non-transcriptional mechanisms, which is a survival-optimized strategy for rapid analgesia during immune challenges (71).

#### Bone marrow

The ontogeny of immune cells traces back to hematopoietic stem cells (HSCs), with bone marrow serving as the principal site of hematopoiesis in postnatal mammals. Sympathetic innervation constitutes the dominant neuronal regulatory network within marrow stroma, critically modulating lymphoid and myeloid lineage differentiation (72). Beyond canonical sympathetic control over the HSC niche (73–75), emerging evidence highlights nociceptive fibers as essential collaborators in stress-induced HSC mobilization and synergistic maintenance of HSC retention. Mechanistically, nociceptors release CGRP that engages the receptor activity modifying protein 1/calcitonin receptor-like receptor (RAMP1/CALCRL) receptor complex on HSCs, triggering Gαs-mediated adenylate cyclase activation and subsequent cyclic adenosine monophosphate (cAMP) elevation to facilitate marrow egress during granulocyte colony-stimulating factor (G-CSF)-mediated mobilization (55).

#### Spleen

As the predominant lymphoid organ, the spleen orchestrates critical immunological functions including immunomodulation, extramedullary hematopoiesis, and inflammatory regulation (76). Neuroimmune mapping studies reveal dual autonomic regulation of splenic activities (77–79), comprising classical sympathetic inputs from celiac ganglia-derived noradrenergic fibers that modulate immune cell trafficking (80). Notably, cutting-edge neuroanatomical investigations have identified specialized nociceptive projections forming vascular-associated and follicular neural networks within splenic parenchyma (35). These DRG-originating sensory fibers employ a conserved CGRP-mediated signaling mechanism through CALCRL/RAMP1 receptor complexes to potentiate antigen-specific antibody production while maintaining immune tolerance. This neural-immune crosstalk offers therapeutic potential for modulating vaccination efficacy and rectifying pathogenic autoantibody responses in B cell-mediated disorders.

#### Lymph nodes

As pivotal immunological filtering centers, lymph nodes (LNs) coordinate the surveillance and processing of peripheral lymph drained via afferent lymphatic channels. Neuroanatomical investigations across mammalian species have identified dual noradrenergic and peptidergic neural networks within LNs (81, 82). Emerging evidence proposes that sensory fibers may regulate regional immunity by altering lymphocyte migration dynamics (81, 83, 84). Cutting-edge multi-omics approaches have unveiled a specialized subset of LN-innervating neurons, predominantly characterized as peptidergic nociceptors through transcriptional profiling. Complementary single-cell RNA sequencing analyses mapped their cellular interactome, revealing robust neuro-stromal crosstalk with endothelial cells, fibroblastic reticular cells, and innate immune populations. These findings delineate the molecular signature of previously unrecognized sensory neurons and elucidate a neuron-stromal signaling axis critical for LN homeostasis.

In summary, the intricate interplay between sensory neurons and the immune system is garnering growing recognition for its profound significance. They act like the body’s “dual alarm system” : both recognize harmful substances and cooperate to protect the body’s tissues and maintain internal stability. It is particularly noteworthy that sensory nerves not only transmit pain sensation but also strengthen the body’s defense by regulating the activity of immune cells. A deeper understanding of the interaction between these two systems will offer new perspectives for disease prevention and treatment, facilitating the development of more targeted therapies.

### Skin system

As one of the important barriers between the body and the external environment, the skin system has two physiological functions: one is to maintain the integrity of the physical barrier to prevent pathogen invasion; the second is to decode environmental information through the perceptual unit to initiate adaptive responses. Recent findings suggest that the sensory nervous system plays an important regulatory role in skin immune defense mechanisms and tissue regeneration.

From the perspective of neuroanatomy, cutaneous nerve fibers are mainly afferent fibers (85), and their neural networks form multi-level projections among the epidermis, dermis, and subcutaneous adipose tissue (86). Histological features analysis showed that nerve fibers were mainly enriched in the middle part of the dermis and the papillary layer region, while the epidermal basement membrane zone, skin vascular network, and accessory microenvironment (such as hair follicles, sebaceous glands, etc.) showed specific distribution characteristics of sensory nerves (87). The structural and functional classification of cutaneous sensory nerves has been systematically described by Winkelmann (88).

#### Skin host defense

Sensory neurons are able to sense the presence of microorganisms and actively participate in skin immunity. Cutaneous immune responses are typically initiated following damage to the epithelial barrier due to microbial invasion or allergen penetration, at which point the pathogen makes direct contact with nociceptive neurons through pattern recognition receptors to elicit pain (89, 90). However, sensory neurons in the skin, such as TRPV1^+^ neurons, can also directly sense pathogens or their associated proinflammatory cytokines, and play a crucial role in the innate defense against skin pathogens.

Nociceptive neurons express a number of receptors that recognize microorganisms, including formyl peptide receptors and TLRs. For example, lipopolysaccharide (LPS), a component of the cell wall of Gram-negative bacteria, binds to the classical receptors TLR4 and CD14 on TRPV1^+^ sensory neurons (91). LPS can also directly act on transient receptor potential ankyrin 1 (TRPA1) channels on sensory neurons, resulting in intraneuronal calcium influx, calcitonin gene-related peptide release and triggering local skin edema (92). Functionally, sensory neurons interact with pathogens to suppress local inflammation. In response to bacterial stimulation, Nav1.8^+^ nociceptive neurons release CGRP, which subsequently acts on macrophages to inhibit tumor necrosis factor α (TNFα) production and ultimately the recruitment of neutrophils and monocytes to the site of infection (57). In addition, TRPV1^+^ sensory neurons are directly activated by the secreted perforin toxin streptococcin S (SLS) after streptococcal infection, and activated TRPV1^+^ sensory neurons can release CGRP, which subsequently inhibits neutrophil recruitment and bactericidal activity (58). It has also been suggested that the activation of TRPV1^+^ sensory neurons can trigger a local type 17 immune response, which not only enhances the body’s defense against Candida albicans and Staphylococcus aureus but also enhances the defense ability of adjacent unstimulated skin through neural reflex arc (59).

#### Wound healing

DRG sensory neurons promote wound healing by regulating neuroimmunity in a manner illustrated in **Figure 3**. For example, sensory neurons expressing the Gαi-interacting protein (GINIP) can produce the neuropeptide TAFA4 to regulate IL-10 production by dermal macrophages, thereby maintaining the survival and function of dermal macrophages, ultimately reducing skin inflammation and promoting tissue regeneration. In contrast, ablation of GINIP^+^ sensory neurons leads to defects in tissue regeneration and dermal fibrosis (56). In addition, CGRP released by sensory neurons stimulates thrombospondin-1 (TSP-1) expression via the RAMP1 receptor on the surface of macrophages and neutrophils (9). This neuroimmune response not only significantly improves the clearance efficiency of neutrophils, but also promotes the transformation of macrophages into a pro-repair phenotype and accelerates the process of tissue repair. Studies have shown that neuroimmunomodulatory mechanisms accelerate the transition of inflammatory response to anti-inflammatory and repair stages, thereby promoting the process of wound healing.

### Respiratory system

The lung receives innervation from various nerves (93). It has been established that the sensory innervation in the lung is mainly from the VG, with minor from the DRG (27, 94).

Breathing is the basic function of the respiratory system which is tightly regulated by the sensory system. Among them, vagal sensory nerves are the major fibers that innervate the lung. Research has demonstrated that a specific subset of vagal afferent neurons plays a crucial role in regulating breathing. When purinergic receptor P2Y1^+^ (P2ry1) neurons in the vagus nerve are optogenetically activated, this activation immediately halts respiration, causing animals to remain in the exhalation phase. Conversely, optogenetic stimulation of neuropeptide Y receptor Y2^+^ (Npy2r) neurons in the vagus nerve leads to rapid and shallow breathing patterns (95). In addition, the afferent vagus nerves, which also innervate the airways, are critical for triggering airway protective mechanisms such as bronchospasm and the cough reflex (96–98).

Besides breathing and airway protective mechanisms, in the lung, neuroimmune interactions especially sensory neurons can help to preserve protective barrier functions while simultaneously curbing pathological inflammation (11, 12, 99). For example, the expression of janus kinase 1 (JAK1) in vagal sensory neurons helps to inhibit airway inflammation. Specifically, a neuronal JAK1-CGRPβ signaling axis can suppress the functions of group 2 innate lymphoid cells (ILC2s) and thereby reduce allergic lung inflammation (61). Besides CGRP, sensory neurons also release other neuropeptides, such as vasoactive intestinal peptide (VIP), and neuromedin U (NMU) to modulate inflammation (11, 100, 101). In addition, in pathological conditions such as *S. aureus*-induced lethal pneumonia, TRPV1^+^ nociceptive neurons in the lung can limit antibacterial immune responses through the action of CGRP (63). These fibers can reduce inflammation and inhibit the recruitment of immune cells. It is still unclear whether distinct neuroimmune pathways regulate different types of lung inflammation, such as that caused by viral infections or fibrosis.

In addition, some reports on the relationship between sensory neurons and other lung diseases, such as pulmonary fibrosis. TRPV1^+^ sensory neurons expressing Toll-like receptors (TLR2 and TLR5). These neurons have been implicated in bleomycin-induced lung fibrosis and associated chronic cough symptoms (102). In contrast, CGRP released by sensory neurons has been shown to limit fibrosis by modulating fibroblast and macrophage activation (103, 104), indicating a potential protective role.

Overall, sensory neurons, particularly those originating from the VG, are primarily crucial for regulating breathing and modulating immune homeostasis in the lung. Besides these local functions, it is widely recognized that pathogen infection induces a typical state of sickness. Recent studies have shown that eliminating petrosal gamma-aminobutyric acid type A receptor subunit α 1-positive (GABRα1^+^) neurons or specifically knocking out the prostaglandin E2 receptor 3 can prevent influenza-induced reductions in food intake, water intake, and mobility during the early stages of infection. Moreover, these interventions improve survival rates. This finding highlights a key airway-to-brain sensory pathway that detects locally produced prostaglandins and mediates systemic sickness responses to respiratory viral infections (105). In addition, one study revealed the complete neural conduction circuit of allergic reactions in the lungs (99). When the respiratory tract is stimulated by persistent allergens, the pathological signal is first mediated by immune cells and then transmitted by the vagus sensory afferent fibers to the dopamine-β-hydroxylase (DBH) positive nucleus of the solitary tract integration center in the brainstem. This center then constructs a descending pathway through adrenergic Adra1a+/Adra1b+ efferent neurons, which ultimately act on postganglionic neurons and regulate airway smooth muscle tone (106).

### Digestive system

The digestive system is a series of organs responsible for converting food into energy and nutrients for the body. The extrinsic sensory nerve terminals in the gastrointestinal (GI) tract are located in the NG, T10-L1 DRG and L4-S1 DRG (107–109).

#### Sensory neurons participate in gastrointestinal dilation response, regulating the transport of gastrointestinal contents

First, the gastrointestinal dilation response requires sensory neurons. Five genetic subtypes of DRG sensory afferents innervated the distal colon and exhibit distinct physiological properties in response to colonic dilation (110). Piezo2 is a rapidly adapting mechanosensitive ion channel expressed in sensory neurons of the DRG and is also expressed in TRPV1-lineage nociceptors (111). Recent studies have demonstrated that Piezo2^+^ sensory neurons play a crucial role in mediating high-threshold colonic distension responses. Functional silencing of TRPV1^+^ sensory neurons significantly attenuates the visceromotor nociceptive responses evoked by colorectal distension, which are mediated by Piezo2^+^ sensory neurons (112).

Second, sensory neurons are involved in the satiety response after GI dilation. Piezo^+^ neurons are mechanically sensitive to sense GI distension signals and rapidly transmit direct satiety signals to the brain to inhibit further feeding (113). Vagal afferents innervate the gut to form Intraganglionic laminar endings (IGLEs), which sense intestinal stretch when the gut is distending and activate satiety-promoting pathways in the brain stem. And by inhibiting AgRP^+^ neurons in the hypothalamus (hunger-promoting agouti-related peptide), ultimately inhibiting food intake (42).

Third, sensory neurons are involved in intestinal peristalsis. The lack of Piezo2 in sensory neurons accelerates gastric emptying and intestinal transport, and Piezo2 knockout mice exhibit diarrhea in bead-expulsion assays, suggesting that Piezo2^+^ sensory neurons are important factors involved in normal intestinal motility (110, 114).

#### Sensory neurons are involved in the process of intestinal pathogen infection, either inhibiting or promoting it

Some studies suggest that gut-innervated nociceptors can defend against intestinal pathogens and promote tissue protection (13, 49). The dorsal root ganglion nociceptor exhibits resistance against the bacterial pathogen Salmonella enterica serovar Typhimurium (STm) during intestinal engraftment and attack. The mechanism is as follows: CGRP is released from dorsal root ganglion nociceptors. It regulates the number of microfold (M) cells in the ileum Peyer’s patch (PP) follicle-associated epithelia and segmentous filamentous bacteria (SFB). As a result, it limits the invasion of STm. The administration of subcutaneous resiniferatoxin (RTX) resulted in a significant reduction of CGRP levels in the DRG and vagus nerve, and pathogen resistance significantly decreased in mice (13). Through viral or pharmacological ablation of TRPV1^+^ sensory neurons, it has been observed in a mouse model of intestinal inflammation that there is an exacerbation of inflammation and impairment in tissue repair processes. This can be attributed to the reduced release of substance P by damaged sensory neurons, which limits the protective effects on intestinal inflammation (115, 116).

But some studies also suggest that sensory neurons promote pathogen infection in the intestinal tract, sensory neurons play an indispensable role in the process of neurogenic inflammation. Sensory neurons can release pro-inflammatory neuropeptides such as substance P (SP) and CGRP, which lead to vasodilatation, increased permeability, inflammatory cell extravasation, tissue edema, and other inflammatory manifestations (44, 48, 117). For example, toxins such as toxin B (TcdB) released after Clostridium difficile infection can stimulate neurons to secrete SP, CGRP and pericytes to secrete pro-inflammatory cytokines, which ultimately promote the development of colonic inflammation. Blocking SP and CGRP signal transduction can reduce the severity of infection (14). There is also a hypothesis that CGRP released after activation of Trpv1^+^ sensory neurons binds to the RAMP1 receptor on regulatory T cells (Tregs) in the gut and inhibits the proliferation of Tregs in the gut, thereby affecting the intestinal inflammatory response (64).

Furthermore, intestinal inflammation can disrupt the regulatory role of gut sensory neurons in intestinal function. These neurons directly perceive inflammatory signals and may initiate self-destruction (ferroptosis) through specific molecular pathways (e.g., interferon-mediated mechanisms), resulting in abnormal gut motility (118). Pharmacological inhibition of these pathways could potentially preserve neuronal integrity and ameliorate post-inflammatory complications.

#### Sensory neurons involved in gut-brain axis mediated behavior regulation, regulating gastrointestinal steady state

**Figure 4:**
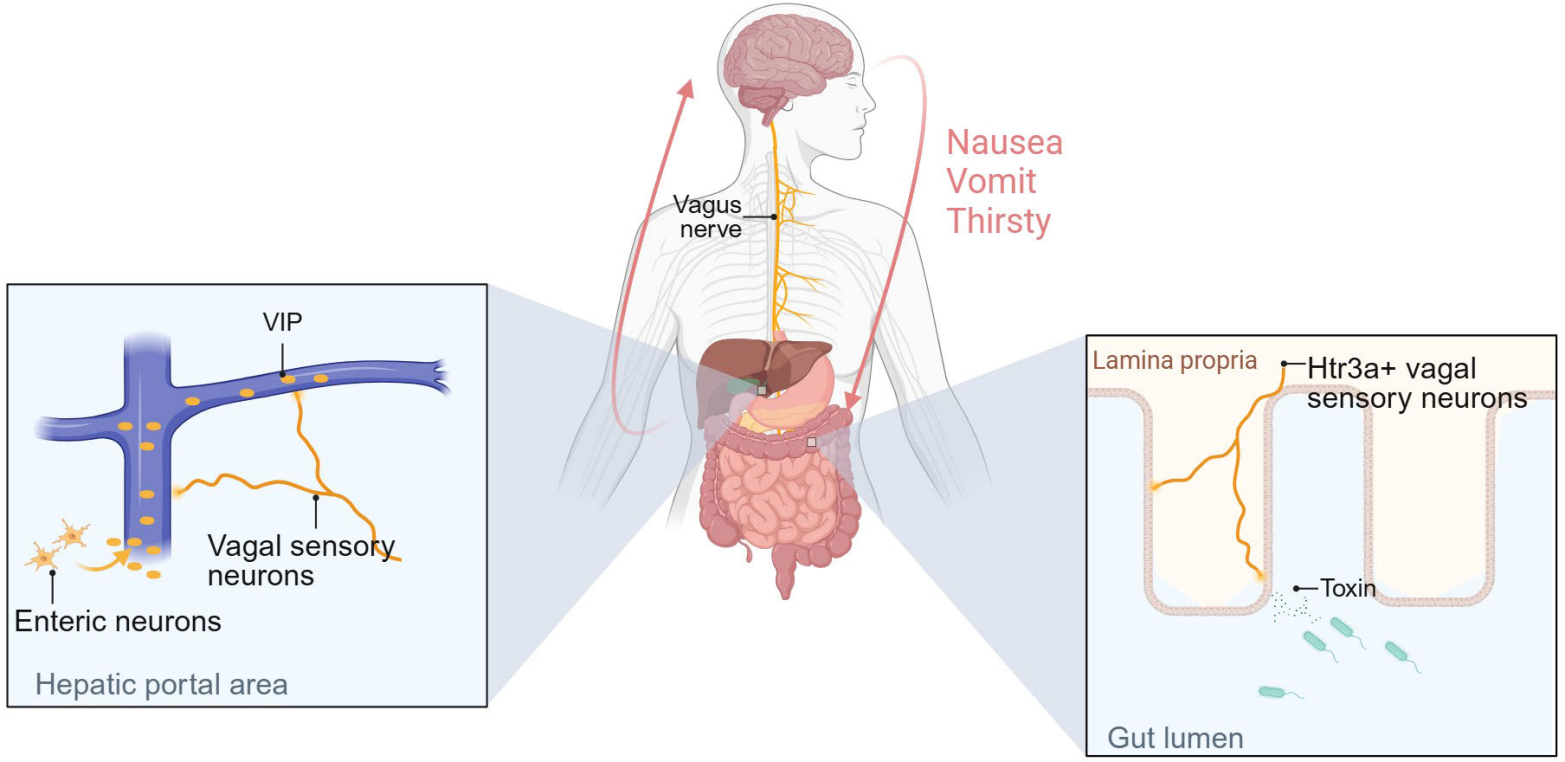
Sensory neurons are involved in gut-brain axis-mediated behavioral regulation and regulate gastrointestinal homeostasis. The figure illustrates the mechanisms by which two types of vagal sensory afferents regulate behavior. One mechanism is as follows: Changes in osmotic pressure in the gut mediate VIP secretion by intestinal neurons, and high concentrations of VIP in the portal vein activate vagal sensory afferents innervating the HPA region, thereby regulating thirst. The other is that Htr3a^+^ vagal sensory neurons can detect toxins secreted by intestinal bacteria and transmit signals to the brain, causing nausea and vomiting. Created with BioRender.com.

Recently, the significance of the gut-brain axis mediated by sensory afferents has been recognized. The liver-brain-gut reflex axis consists of hepatic vagal afferent, nucleus tractus solitarius and enteric neurons. This axis maintains intestinal homeostasis by regulating the differentiation and maintenance of peripheral regulatory T cells (pTreg cells). Disruption of afferents to this axis results in increased susceptibility to colitis (119). 5-hydroxytryptamine receptor 3A positive-(Htr3a^+^) vagal sensory neurons can detect intestinal bacterial toxins and transmit signals to tachykinin precursor 1 positive-(Tac1^+^) neurons in the dorsal complex (DVC) via the vagus nerve, ultimately leading to nausea and retching behavior (120). Another intriguing study has demonstrated that ingested water rapidly suppresses thirst through osmoreceptors before being absorbed into the gut. Specifically, the hypotonic signal of ingested water stimulates enteric neurons to release VIP, which activates the vagus nerve innervating the hepatic portal area (HPA) and regulates thirst via vagal afferents to the brain (121). These mechanisms are summarized in **Figure 4** (**Figure 4**). In addition, the vagus nerve is involved in the transmission of pain signals in the stomach-brain axis, transmitting the gastric nociceptive signal to the first level center: nucleus tractus solitarius (NTS) (54).

### Metabolism system

The nervous system regulates metabolic activities of the body through neurotransmitters, neuroendocrine cells, and neural networks. Research on sensory nerves in metabolism has focused more on adipose tissue (122–126). Innervation in adipose tissue is predominantly sympathetic and sensory. Sympathetic nerves mainly release norepinephrine, while sensory nerves mainly release neuropeptides such as CGRP and SP. The two coordinate with each other and participate in the fat metabolism network (127, 128).

#### Brown adipose tissue

Brown adipose tissue is an important thermogenic organ. CGRP secreted by sensory nerves coordinates with sympathetic nerves to maintain the stability of thermogenesis and adapt to environmental temperature changes (127, 128). When the body senses a low temperature, sympathetic nerves distributed in brown adipose tissue are activated, resulting in the release of norepinephrine, which eventually acts on the mitochondrial membrane transmembrane protein uncoupling protein 1 (UCP1) in brown adipose tissue, and UCP1 generates heat through uncoupling (129). It was found that injection of the sensory neurotoxin capsaicin into the interscapular adipose tissue of Siberian hamsters destroyed sensory nerves, resulting in decreased UCP1 protein levels, reduced brown adipose tissue mass, and impaired thermoregulation (reduced thermogenic effect in response to acute cold) (128, 130). In another study, intravenous norepinephrine was used to mimic the thermogenic effect in rats, whereas sensory denervation of brown adipose tissue with capsaicin prolonged noradrenaline-induced thermogenesis. It is noteworthy that intravenous CGRP mimics sensory nerve activation while reducing the thermal effect induced by norepinephrine. This indicates that sensory nerves in brown adipose tissue are likely to form a feedback loop with sympathetic nerves, coordinating sympathetic neurotransmitter thermogenesis, promoting sympathetic thermogenesis when ambient temperature or local tissue temperature is too low, and inhibiting sympathetic thermogenesis when the temperature is too high, thus adapting to ambient temperature (131, 132).

#### White adipose tissue

White adipose tissue is a storage form of energy. Different from brown adipose tissue, sensory nerves in white adipose tissue are more involved in regulating the synthesis and decomposition of fat, thereby regulating the homeostasis of fat metabolism, and this may be achieved by inhibiting sympathetic nerves. After sensory denervation with capsaicin, the expression of CGRP in white adipose tissue was decreased (indicating successful sensory nerve ablation), but the adipocytes became hypertrophic (127), indicating that lipolysis was inhibited after sensory nerve ablation. Sensory denervation with capsaicin also promoted the proliferation and differentiation of white adipocytes (133). Injection of ROOT-Cre adeno-associated virus into subcutaneous inguinal adipose tissue to ablate the specific sensory projections of adipose tissue resulted in increased markers of new adipose tissue (fatty acid synthase, acetyl CoA carboxylase β) and increased fat content (134).

#### Maintenance of metabolism

The sensory nerve plays a crucial role in regulating the body’s normal metabolic function, ensuring stable control of blood sugar levels and metabolism rate, thereby preventing metabolic disorders. It has been shown that glucagon-like peptide 1 receptor-positive (GLP1R^+^) vagal afferent activation in the gut increases glucose uptake in skeletal muscle and sends anorexigenic signals to the brain stem, thereby lowering blood glucose. In contrast, G protein-coupled receptor 65-positive (GPR65^+^) vagal afferent activation promotes hepatic glucose production via phosphoenolpyruvate carboxykinase 1 (Pck1), which raises blood glucose levels. This implies that GLP1R^+^ and GPR65^+^ sensory neurons work together to maintain blood glucose balance and the metabolic homeostasis of the body (135–137).

### Cancer

Under physiological conditions, the sensory neurons are vital for tissue homeostasis. They detect diverse stimuli and transmit signals. The PNS releases neurotransmitters/neuropeptides like CGRP, which regulate blood vessels and immune cell extravasation. It also modulates tissue repair by influencing stem cells and the inflammatory response at injury sites. However, sensory neurons are also involved in tissue homeostasis imbalances, such as tumors. Previous studies have shown that solid tumors can be innervated by autonomic and sensory nerve fibers (138–140). In recent years, the mechanisms and effects of the interaction between solid tumors and nerves have been gradually revealed, especially in the crosstalk between sensory neurons and solid tumors.

**Figure 5:**
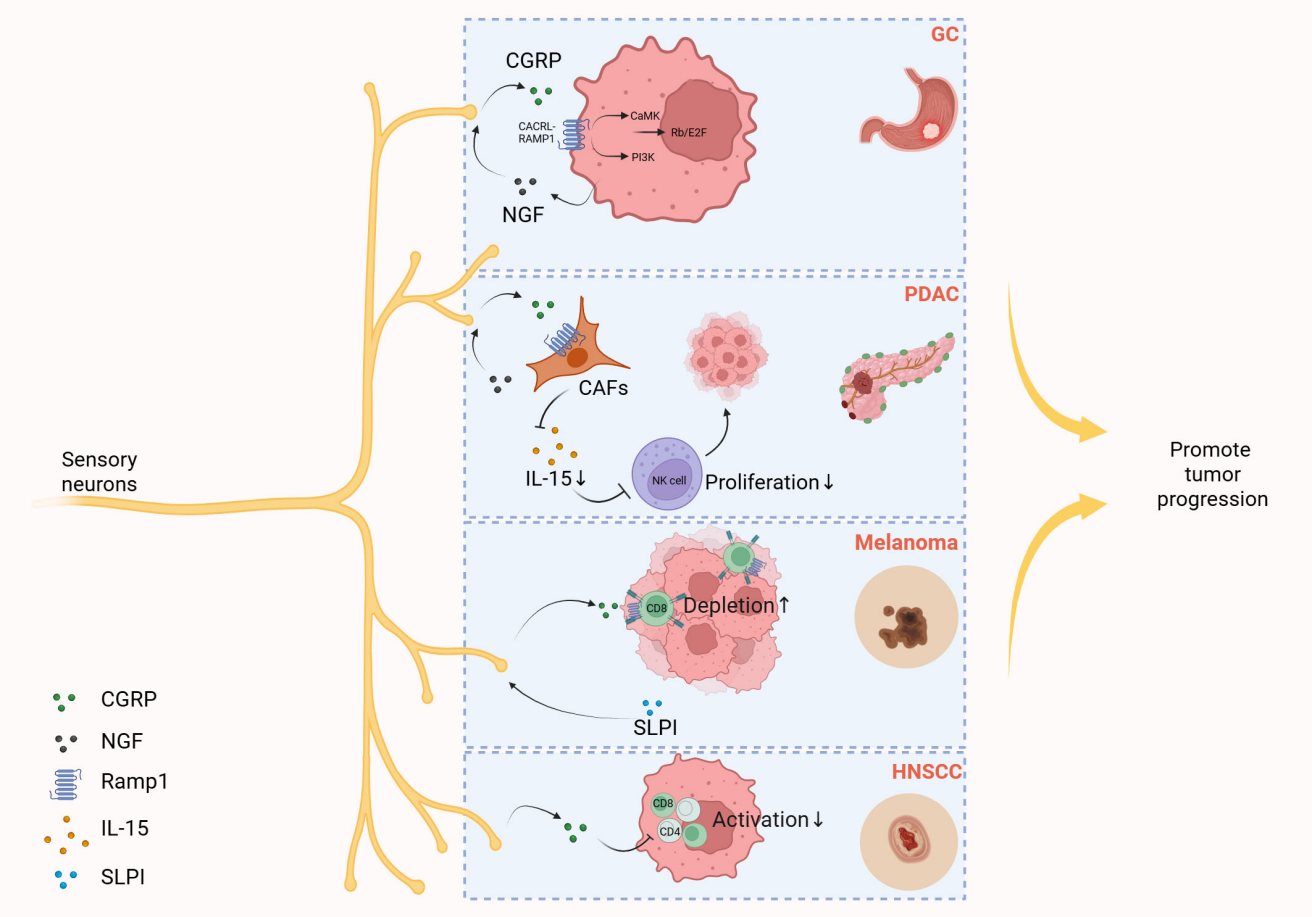
Sensory neurons promote tumor progression by releasing CGRP. Gastric cancer (GC) cells elevate the secretion of nerve growth factor (NGF), and then NGF induces CGRP release from sensory neurons. CGRP then engages the Calcrl/Ramp1 on gastric cancer cells, triggering dual activation of PI3K-Akt and CaMK-dependent signaling cascades. These pathways converge to amplify E2F-dependent transcriptional programs, fostering tumor progression through cell cycle deregulation and survival mechanisms. In the tumor microenvironment of pancreatic ductal adenocarcinoma (PDAC), cancer-associated fibroblasts (CAFs) secrete NGF that stimulates sensory neurons to release CGRP. This neuropeptide subsequently binds to receptor RAMP1 on CAFs, triggering downregulation of interleukin-15 (IL-15) expression. The reduced IL-15 levels impair NK cell infiltration and cytotoxic activity, ultimately fostering tumor progression in PDAC. In melanoma, tumor-derived secretory leukocyte protease inhibitor (SLPI) induces pain hypersensitivity and stimulates nociceptor neurite outgrowth and sustained release of CGRP, which binds to RAMP1 expressed on tumor-infiltrating CD8^+^ T cells, triggering upregulation of immune checkpoint receptors, driving functional exhaustion of cytotoxic CD8^+^ T cells, resulting in uncontrolled melanoma cell proliferation. In the microenvironment of head and neck squamous cell carcinoma (HNSCC), CGRP released by sensory neurons can directly suppress immune cells, namely T helper 1 (Th1) CD4^+^ T cells and activated CD8^+^ T cells, thereby promoting tumor growth. Created with BioRender.com.

It is noteworthy that sensory neurons are likely involved in tumorigenesis and progression, primarily through the release of CGRP and SP (141, 142). For instance, a recent study revealed that sensory neurons promote gastric tumor development and metastasis via the CGRP-RAMP1 axis (143). As research progresses in exploring the regulatory mechanisms of sensory neurons in tumors, evidence suggests that CGRP released from sensory neurons may facilitate tumorigenesis by suppressing immune regulation within the tumor microenvironment. Ru-Rong Ji et al. demonstrated that sensory neuron-derived CGRP interacts with cancer-associated fibroblasts to inhibit the infiltration and cytotoxic function of natural killer (NK) cells, ultimately promoting pancreatic ductal adenocarcinoma progression (144). Sebastien Talbot et al. found that CGRP released by sensory neurons promoted the depletion of cytotoxic CD8^+^ T cells, thereby limiting their ability to eradicate melanoma. Conversely, inhibition of CGRP expression or antagonism of the CGRP receptor reduced the depletion of leukocytes in tumors, and finally, the tumor growth was inhibited and the survival rate of mice was improved (141). In addition, Laurel Darragh et al. found that CGRP released from sensory neurons can directly inhibit immune cells (Th1 CD4^+^ T cells and activated CD8^+^ T cells) in the environment of head and neck squamous cell carcinoma (HNSCC), thereby promoting tumor growth (15). Here, we summarize the mechanisms by which CGRP released from sensory neurons regulates tumor progression, as shown in **Figure 5** (**Figure 5**).

Other mechanistic pathways have also been identified. In breast cancer, sensory nerves release the neuropeptide SP, which binds to tachykinin receptor 1 (TACR1) on cancer cells, inducing cell death and the release of single-stranded RNA (145). Another study revealed that deletion of the tumor suppressor gene TP53 causes microRNA miR-34a to shuttle from cancer cells to tumor-associated sensory neurons, driving the conversion of these neurons to an adrenergic phenotype. Such crosstalk between cancer cells and neurons promotes tumor development. Conversely, sensory neuron denervation or blockade of adrenergic signaling inhibits tumor growth, highlighting a potential target for cancer therapy (142).

### Cardiovascular system

Research on sensory nerves in cardiovascular regulation focuses primarily on blood vessels, especially peripheral vessels. Sensory neurons mainly release CGRP and SP to participate in vascular dilatation. In addition, sensory neurons also participate in the differentiation of vascular smooth muscle with weak contraction function and promote angiogenesis. Many studies have also suggested that sensory neurons are involved in the regulation of cardiac structure and function during heart diseases (146–148).

#### Blood pressure

In general, sensory nerves maintain the stability of blood pressure in the body. CGRP, a neuropeptide released by sensory neurons, binds to vascular CGRP receptors, inducing vasodilation and subsequent blood pressure reduction (149, 150). Evidence also suggests that CGRP regulates blood pressure by indirectly inhibiting sympathetic-mediated vasoconstriction (151, 152). In addition, it has been found that non-peptide sensory neurons can also regulate arterial blood pressure. Tropomyosin receptor kinase C positive (TrkC^+^) sensory neurons in the spinal ganglia can project to the distal artery, and local stimulation of TrkC^+^ sensory neurons can reduce blood vessel diameter and blood flow, and can rapidly increase blood pressure after chronic activation of this type of neurons (153).

#### Angiogenesis

Sensory neurons promote endothelial cell proliferation and participate in angiogenesis by secreting neuropeptides. *In vitro* studies show that sensory neurons enhance angiogenesis through two mechanisms: secreting CGRP and SP to promote extracellular matrix remodeling in endothelial cells. Specifically, CGRP upregulates vascular endothelial growth factor A (VEGF-A) expression, while SP upregulates angiopoietin 1 (Angpt1), collagen type IV (Col IV), and matrix metalloproteinase 2 (MMP2) expression (147). Notably, these genes are all angiogenic markers in endothelial cells. In addition, the model of dry eye disease was established through inflammatory stimulation, and then trigeminal ganglion sensory neurons were isolated and co-cultured with corneal vascular endothelial cells. Compared with the control group, sensory neurons stimulated by inflammation expressed and secreted more SP and promoted the proliferation of vascular endothelial cells (154). It has also been found that CGRP secreted by sensory neurons of a trigeminal ganglion can promote corneal angiogenesis and lymphangiogenesis *in vivo*, and subconjunctival injection of CGRP antagonist CGRP8–37 reduces secretion-induced corneal angiogenesis (148). These data suggest that sensory neurons are involved in the formation of blood vessels by secreting neuropeptides.

#### Heart

Sensory neurons are also involved in the regulation of cardiac structure and function. Firstly, sensory neurons in the heart are involved in the cardiac reflex: the Bezold-Jarisch reflex (BJR) (155). BJR is a cardiac depression reflex characterized by the classic triad of hypotension, bradycardia, and respiratory depression (156, 157). Recent studies have shown that vagal neurons expressing neuropeptide Y receptor Y2 (NPY2R) are required for cardiac BJR: when these sensory neurons are activated, they induce BJR responses and cause syncope in animals. Secondly, TRPV1 receptors on cardiac sensory neurons have garnered significant attention. When TRPV1^+^ sensory neurons are activated by injury, inflammation and other factors, they can release CGRP, SP and pituitary adenylate cyclase-activating polypeptide (PACAP). These neuropeptides can induce vasodilation and inflammatory cell activation (158–160). In addition, several studies have shown that TRPV1^+^ sensory neurons are involved in atherogenesis and play an important role in structural remodeling caused by acute myocardial infarction and heart failure (161–164). Recent studies have also found that Piezo1 in thoracodorsal root ganglion (TDRG) neurons mediates a neuroimmune response that exacerbates ventricular remodeling after myocardial infarction (165).

## Cellular and molecular cross-talking coordination between DRG/VG with organs

**Figure 6:**
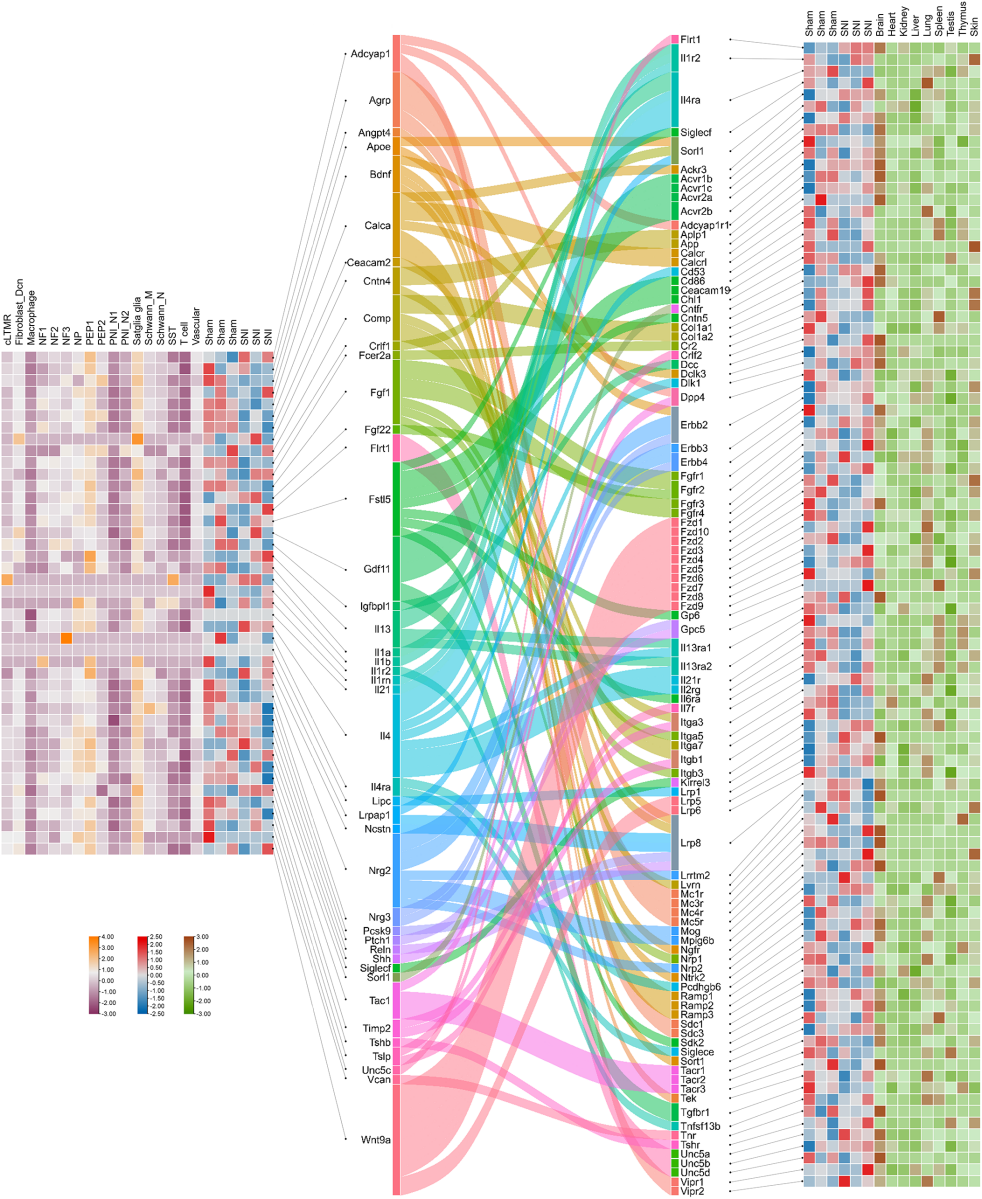
Crosstalk between ligands secreted by cells in the mouse dorsal root ganglia and receptors in the organs. Heatmap of ligands that were identified by single nucleus RNA sequencing and bulk-seq analysis describes the expression levels of ligands within the cell clusters and dorsal root ganglia in both sham and SNI states. Sankey diagram reveals the ligands and the corresponding receptors predicted by Cellinker. Heatmap of receptors describing the expression levels of receptors within dorsal root ganglia in both sham and SNI states and different organs of an adult male C57BL/6 mouse (166).

To investigate the interaction between ligands secreted by mouse DRG cells and receptors in peripheral organs, we leveraged single-nucleus RNA sequencing (snRNA-seq) data to analyze cell-cell interactions based on ligand expression profiles of DRG cells. Using Cellinker, a computational tool for predicting ligand-receptor (L-R) gene crosstalk, we identified 123 valid L-R pairs, comprising 43 ligands and 98 receptors, between DRG and various organs. Among the identified ligands, the *Calca* gene, encoding CGRP, and the *Tac1* gene, encoding SP, were of particular interest, as both CGRP and SP are secreted by sensory neurons to mediate various physiological functions. Our analysis uncovered several neuropeptide-related L-R pairs, including *Calca_Ackr3*, *Calca_Calcr*, *Calca_Calcrl*, *Calca_Dclk3*, *Calca_Ramp1*, *Calca_Ramp2*, *Calca_Ramp3*, *Tac1_Dpp4*, *Tac1_Tacr1*, *Tac1_Tacr2*, and *Tac1_Tacr3*. Notably, these ligands are primarily secreted by peptidergic nociceptors (PEP1/PEP2 subsets) and satellite glial cells within the DRG, while their corresponding receptors are expressed in various organs, predominantly in the brain, kidneys, and lungs. This suggests that these L-R interactions are likely involved in the regulation of organ-specific functions by sensory neurons (**Figure 6**).

To assess how cellular communication in the DRG is altered under different conditions, we also analyzed differentially expressed ligand and receptor genes using bulk RNA sequencing (RNA-seq) data from both normal and sciatic nerve injury (SNI) conditions. Our results showed that, after SNI, both *Calca* and *Tac1* were down-regulated in the DRG, and corresponding changes were observed in the expression of their receptors. Specifically, *Calca_Ramp2*, *Tac1_Tacr1*, and *Tac1_Tacr2* were significantly down-regulated under injury conditions. Additionally, we identified several genes, including *Flrt1*, *Il1r2*, *Il4ra*, *Siglecf*, and *Sorl1*, that act as both ligands secreted by DRG cells and receptors expressed in target organs. These dual functions underscore the complexity of DRG-mediated signaling in organ regulation.

**Figure 7:**
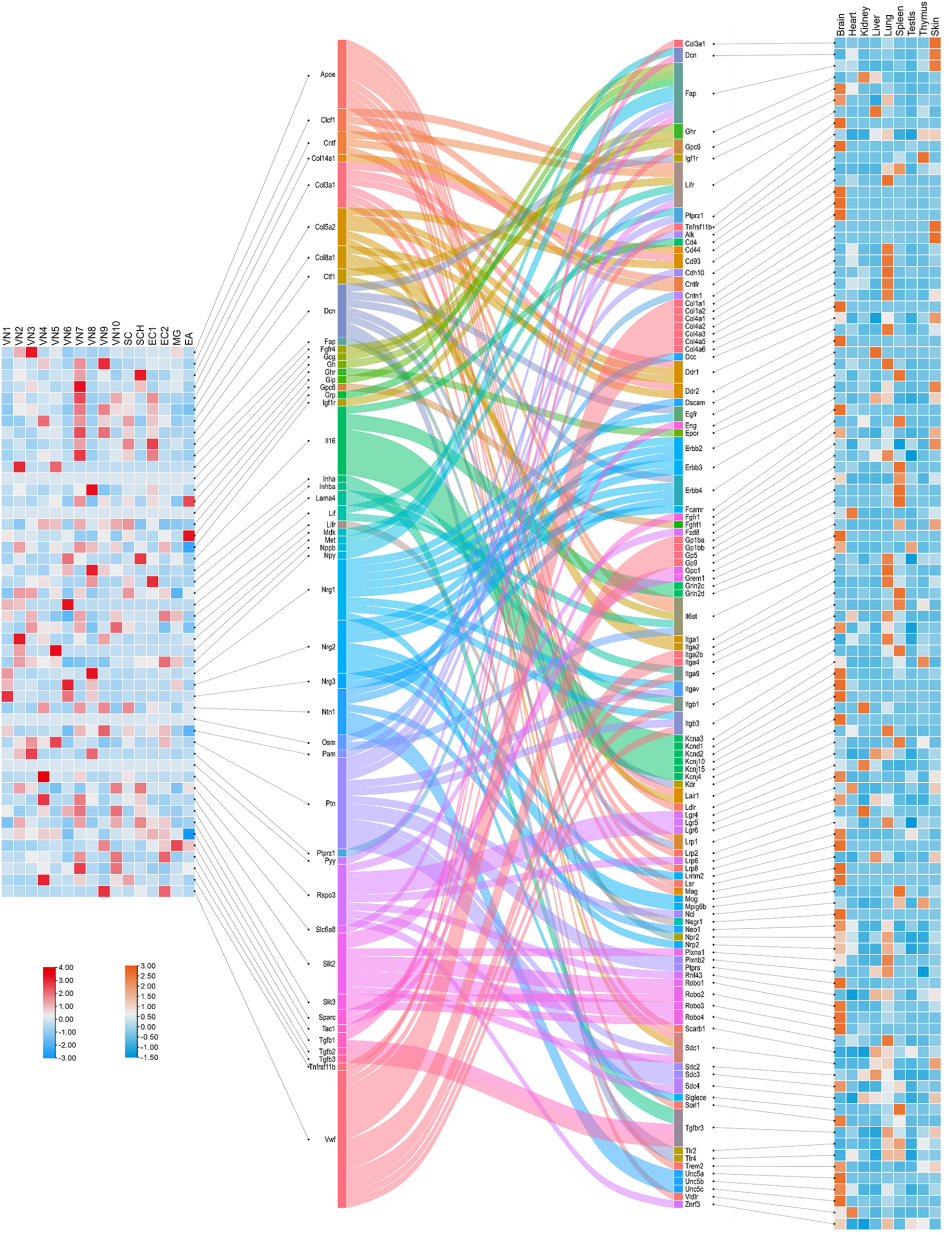
Crosstalk between ligands secreted by cells in the mouse vagal ganglia and receptors in the organs. The left heatmap shows the expression levels of ligands across cell clusters within the vagal ganglia, based on single-nucleus RNA sequencing (snRNA-seq) data from Forster et al (167). The central Sankey diagram visualizes predicted ligand-receptor interactions, as computed using Cellinker, illustrating the diversity of VG-derived signals and their potential targets across tissues. The right heatmap displays the expression levels of corresponding receptors in various organs of an adult male C57BL/6 mouse, derived from reference transcriptomic datasets (166).

To complement the DRG-focused analysis, we next explored ligand-receptor crosstalk originating from the VG, which plays a central role in visceral sensory regulation. Using single-nucleus RNA sequencing (snRNA-seq) data from VG neurons, we assessed cell-cell communication based on ligand expression profiles across neuronal subtypes (**Figure 7**). A total of 153 valid ligand-receptor (L-R) pairs were identified through Cellinker prediction, comprising 48 ligands and 104 receptors that potentially mediate VG-organ interactions.

Ligand expression was mapped across 10 transcriptionally distinct neuronal clusters within the VG, revealing considerable subtype-specific heterogeneity. This layered organization suggests that molecular outputs from VG neurons are finely tuned by cell identity, possibly reflecting differential projections to visceral targets. The corresponding receptor expression data, integrated from peripheral tissues, enabled the construction of a connectivity matrix visualized via a Sankey diagram. This analysis uncovered complex patterns of VG-to-organ communication, with multiple ligands showing convergence onto specific targets, and vice versa.

Notably, several molecules—including *Col3a1*, *Dcn*, *Fap*, *Ghr*, *Gpc6*, *Igf1r*, *Lifr*, *Ptprz1*, and *Tnfrsf11b*—were found to function both as ligands in VG neurons and as receptors in peripheral tissues. These dual-role genes may represent key nodes within bidirectional neuro-visceral circuits. Collectively, this analysis extends our mapping of sensory-derived signaling beyond somatic DRG neurons and highlights the unique and system-specific features of vagal sensory neurobiology.

## Discussion

The expanding understanding of the PNS has revealed its profound influence beyond traditional sensory perception, particularly in the regulation of organ function and systemic homeostasis. Recent advancements in technical tools have allowed for more detailed exploration of the interactions between the PNS and various organs, uncovering a spectrum of functions that extend well beyond the classic sensory roles.

### Newly discovered innervation patterns

Recent anatomical studies have uncovered unexpected patterns of peripheral sensory neuron innervation in organs such as the spleen, kidneys, lymph nodes, and gastrointestinal tract (35, 36, 49, 168). These findings, enabled by tissue clearing and genetic labeling techniques, suggest that sensory neurons are strategically positioned to regulate organ-specific functions. For instance, CGRP^+^ and TRPV1^+^ nociceptors project from thoracic DRGs into B cell zones in the spleen, while Nav1.8^+^ neurons interface with goblet cells in the colon (35). Such close anatomical associations imply direct modulation of immune and epithelial cell activity (10).

However, the functional implications of these precise innervation patterns remain largely unexplored. Do these neurons exert tonic influence, respond only to stress or inflammation, or both? Are there organ-specific differences in signaling modes? Future studies combining functional imaging, optogenetics, and conditional ablation will be critical for determining whether these structural connections translate into dynamic, context-dependent regulation of tissue function.

### Direct activation of the peripheral nervous system by unconscious stimuli

The PNS has garnered increasing recognition for its critical role in detecting and responding to “danger signals” and microenvironmental changes within various organs. This evolving understanding extends the traditional view of the somatosensory system from merely sensing external stimuli to actively monitoring and modulating internal physiological states.

Such direct activation of the PNS is crucial for maintaining organ health and defending against infections, particularly at barrier surfaces like the skin, airways, and gastrointestinal tract, which are frequently exposed to microbial invaders. The somatosensory neurons, especially nociceptors, are well-equipped to sense and respond to these threats due to the wide range of PRRs they express, such as TLRs. These receptors enable nociceptors to directly detect microbial invasion, although nociceptors can also be activated indirectly by microbial products (5). For example, nociceptors are directly activated by pathogens such as *Candida albicans* in the skin, *Salmonella* and *Clostridioides difficile* in the gut, and *Mycobacterium tuberculosis* in the lungs (13, 14, 59, 169). This activation triggers appropriate physiological responses, such as inflammation and immune activation, that are essential for host defense.

However, the activation of nociceptors by microbes is a double-edged sword. While it is crucial for pathogen defense, it can also contribute to the pathogenesis of diseases. For instance, TRPV1^+^ nociceptors in the lungs detect lipopolysaccharides (LPS) from non-biofilm-producing *Pseudomonas aeruginosa* via TLR4, leading to the activation of acute stress neurocircuits in the hypothalamus. This process not only drives sickness behavior but may also exacerbate inflammatory responses, contributing to the disease burden (170).

### Neuropeptides, chemokines/cytokines and neurotransmitters released from the peripheral nervous system to activate cells outside the nervous system

A key discovery in recent years is that peripheral sensory neurons release neuropeptides—such as CGRP and SP—that act on diverse cell types beyond the nervous system. These molecules have receptors widely expressed across organs, enabling them to regulate immune responses, tissue repair, and homeostasis (61). For example, CGRP modulates ILC2 activity in the lung, promotes type 17 immunity in the skin, shapes humoral responses in the spleen, and contributes to gut barrier defense. It also influences hematopoietic stem cell mobilization, mucus secretion, and macrophage polarization in tissue repair. However, CGRP can also impair anti-tumor immunity by promoting CD8^+^ T cell exhaustion, highlighting its context-dependent effects (13, 35, 59).

Similarly, SP regulates intestinal inflammation and microbiota balance, but may also exacerbate gut pathology during infection (49). Other mediators—such as TAFA4, glutamate, and CCL2—further illustrate the complexity of sensory neuron-derived signals in shaping immune and stromal cell behavior (56, 60).

Despite these advances, key questions remain. Why do identical mediators yield opposing outcomes across tissues? How do multiple neuroactive molecules released simultaneously interact to influence physiological or pathological states? Answering these questions will be essential to fully understand the integrated regulatory roles of the PNS.

### The peripheral nervous system acts as afferents to transmit new information to the CNS

The PNS, long recognized for relaying sensory information to the CNS, is now understood to transmit signals reflecting the internal state of organs, including immune and metabolic cues. This afferent role is particularly evident in the visceral sensory system, which supports gut-brain communication essential for maintaining systemic homeostasis.

Recent studies have shown that vagal sensory neurons detect signals from enterochromaffin cells, microbiota, and nutrient content to influence CNS activity. For instance, Htr3a+ neurons mediate toxin-induced nausea, while other vagal subsets modulate gut sympathetic tone or promote dopamine release, affecting gastrointestinal function and behavior (120, 171). Vagal afferents in other organs also play specialized roles: they regulate hunger through intestinal mechanosensation, induce cardiovascular reflexes via NPY2R-expressing neurons, and trigger stress responses during lung infection (155). In the liver, vagal pathways help maintain gut immune balance by monitoring osmotic changes (119, 121).

Notably, even afferents from the same organ exhibit distinct molecular and functional profiles, underscoring the specialization within the PNS. However, how these pathways are organized, integrated centrally, and influenced by disease remains poorly understood. Elucidating their molecular coding and projection logic will be key to understanding how the PNS coordinates body-wide homeostasis.

### Pathological roles and disease involvement

While the PNS plays a vital role in maintaining homeostasis, growing evidence suggests it can also contribute to disease pathogenesis. Neuropeptides such as CGRP, released by sensory neurons, have been implicated in promoting melanoma progression and exacerbating chronic inflammation, highlighting how PNS-mediated immune modulation may under certain conditions favor pathology rather than protection (141, 170). Conversely, in breast cancer, SP released from sensory nerves can induce tumor cell apoptosis, illustrating the PNS’s capacity for both promoting and suppressing disease (145).

This dualistic nature underscores the complexity of PNS involvement in pathological states and the need to understand the context-dependent mechanisms that govern its protective versus harmful roles. Clarifying the molecular and cellular pathways that mediate this shift will be essential for developing therapies that harness the beneficial functions of the PNS while minimizing its contribution to disease.

### Advantages of PNS regulatory functions

The regulatory functions of the PNS offer distinct advantages, particularly in its ability to provide rapid and localized responses to environmental changes. By directly modulating organ function and immune responses, the PNS can swiftly adapt to threats, maintaining homeostasis without necessitating extensive systemic alterations. This capability to fine-tune physiological processes at the local level is vital for survival in a constantly changing environment, underscoring the PNS’s role as a central integrator of bodily functions. Additionally, as an afferent pathway, the PNS transmits critical information about the tissue microenvironment to the CNS, enabling the CNS to make informed systemic and behavioral decisions. This dual function—local regulation and systemic communication—highlights the PNS’s essential role in orchestrating a coordinated response to both internal and external challenges.

## Conclusion

The peripheral nervous system is increasingly recognized as a critical player in the regulation of organ function and systemic homeostasis, with roles that extend well beyond traditional sensory perception. The complex interactions between the PNS and various organs open new avenues for therapeutic interventions, especially in diseases where PNS activity may be dysregulated. Understanding these interactions and the mechanisms by which the PNS influences health and disease is essential for developing innovative treatments. Future research should focus on elucidating the molecular and cellular pathways involved in PNS regulation, to leverage its protective functions while mitigating its potential contributions to pathology. As our knowledge of the PNS expands, so too will our ability to harness its capabilities in the pursuit of improved health outcomes.

## Future directions

Research on the non-sensory functions of sensory neurons will continue to deepen, revealing their potential roles in metabolic regulation, immune response, and tissue repair. In the future, new neuropeptides and receptor-ligand pairs other than CGRP and SP may be found to further expand the understanding of the regulatory mechanism of sensory neurons. The crosstalk between sensory neurons and the immune system is an important research direction. It has been shown that nerve damage or modulation can affect inflammatory responses, such as relieving symptoms of arthritis and inflammatory bowel disease. This suggests the key role of sensory neurons in neuro-immune regulation and may provide a new target for inflammation-related diseases. In addition, sensory neurons may play a role in neurodegenerative diseases, tumors, and metabolic diseases. Specific modulators, such as neuropeptide release regulation and neuronal activation/silencing techniques, are expected to become important directions for future treatment. Non-invasive neuromodulation techniques, such as transcranial magnetic stimulation and ultrasound modulation, have shown the potential to more accurately regulate the function of sensory neurons and promote the development of personalized treatment.

## References

[1] Murtazina A Adameyko I. The peripheral nervous system. Development. (2023) 150:9. doi: 10.1242/dev.201164 37170957

[2] Crawford LK Caterina MJ. Functional anatomy of the sensory nervous system: updates from the neuroscience bench. Toxicol Pathol. (2020) 48:174–89. doi: 10.1177/0192623319869011 31554486

[3] Ran C Boettcher JC Kaye JA Gallori CE Liberles SD. A brainstem map for visceral sensations. Nature. (2022) 609:320–6. doi: 10.1038/s41586-022-05139-5 PMC9452305 36045291

[4] Mehrpour V Martinez-Trujillo JC Treue S. Attention amplifies neural representations of changes in sensory input at the expense of perceptual accuracy. Nat Commun. (2020) 11:2128. doi: 10.1038/s41467-020-15989-0 32358494 PMC7195455

[5] Donnelly CR Chen O Ji RR. How do sensory neurons sense danger signals? Trends Neurosci. (2020) 43:822–38. doi: 10.1016/j.tins.2020.07.008 PMC7530006 32839001

[6] Chen CY Shih YC Hung YF Hsueh YP. Beyond defense: regulation of neuronal morphogenesis and brain functions via Toll-like receptors. J BioMed Sci. (2019) 26:90. doi: 10.1186/s12929-019-0584-z 31684953 PMC6827257

[7] Feng Z Sun R Cong Y Liu Z. Critical roles of G protein-coupled receptors in regulating intestinal homeostasis and inflammatory bowel disease. Mucosal Immunol. (2022) 15:819–28. doi: 10.1038/s41385-022-00538-3 35732818

[8] Li L Acioglu C Heary RF Elkabes S. Role of astroglial toll-like receptors (TLRs) in central nervous system infections, injury and neurodegenerative diseases. Brain Behav Immun. (2021) 91:740–55. doi: 10.1016/j.bbi.2020.10.007 PMC7543714 33039660

[9] Lu YZ Nayer B Singh SK Alshoubaki YK Yuan E Park AJ. CGRP sensory neurons promote tissue healing via neutrophils and macrophages. Nature. (2024) 628:604–11. doi: 10.1038/s41586-024-07237-y PMC11023938 38538784

[10] Yang D Jacobson A Meerschaert KA Sifakis JJ Wu M Chen X. Nociceptor neurons direct goblet cells via a CGRP-RAMP1 axis to drive mucus production and gut barrier protection. Cell. (2022) 185:4190–205.e25. doi: 10.1016/j.cell.2022.09.024 36243004 PMC9617795

[11] Talbot S Abdulnour RE Burkett PR Lee S Cronin SJ Pascal MA. Silencing nociceptor neurons reduces allergic airway inflammation. Neuron. (2015) 87:341–54. doi: 10.1016/j.neuron.2015.06.007 PMC4506220 26119026

[12] Tränkner D Hahne N Sugino K Hoon MA Zuker C. Population of sensory neurons essential for asthmatic hyperreactivity of inflamed airways. Proc Natl Acad Sci U S A. (2014) 111:11515–20. doi: 10.1073/pnas.1411032111 PMC4128113 25049382

[13] Lai NY Musser MA Pinho-Ribeiro FA Baral P Jacobson A Ma P. Gut-innervating nociceptor neurons regulate Peyer’s patch microfold cells and SFB levels to mediate salmonella host defense. Cell. (2020) 180:33–49.e22. doi: 10.1016/j.cell.2019.11.014 31813624 PMC6954329

[14] Manion J Musser MA Kuziel GA Liu M Shepherd A Wang S. C. difficile intoxicates neurons and pericytes to drive neurogenic inflammation. Nature. (2023) 622:611–8. doi: 10.1038/s41586-023-06607-2 PMC11188852 37699522

[15] Darragh LB Nguyen A Pham TT Idlett-Ali S Knitz MW Gadwa J. Sensory nerve release of CGRP increases tumor growth in HNSCC by suppressing TILs. Med. (2024) 5:254–70.e8. doi: 10.1016/j.medj.2024.02.002 38423011 PMC10939743

[16] Sengupta JN. An overview of esophageal sensory receptors. Am J Med. (2000) 108 Suppl 4a:87s–9s. doi: 10.1016/S0002-9343(99)00344-7 10718458

[17] Terrier LM Hadjikhani N Destrieux C. The trigeminal pathways. J Neurol. (2022) 269:3443–60. doi: 10.1007/s00415-022-11002-4 35249132

[18] Lawson SN. Phenotype and function of somatic primary afferent nociceptive neurones with C-, Adelta- or Aalpha/beta-fibres. Exp Physiol. (2002) 87:239–44. doi: 10.1113/eph8702350 29345433

[19] Watson JC Dyck PJ. Peripheral neuropathy: A practical approach to diagnosis and symptom management. Mayo Clin Proc. (2015) 90:940–51. doi: 10.1016/j.mayocp.2015.05.004 26141332

[20] Julius D. TRP channels and pain. Annu Rev Cell Dev Biol. (2013) 29:355–84. doi: 10.1146/annurev-cellbio-101011-155833 24099085

[21] Woolf CJ Ma Q. Nociceptors–noxious stimulus detectors. Neuron. (2007) 55:353–64. doi: 10.1016/j.neuron.2007.07.016 17678850

[22] Baral P Udit S Chiu IM. Pain and immunity: implications for host defence. Nat Rev Immunol. (2019) 19:433–47. doi: 10.1038/s41577-019-0147-2 PMC6700742 30874629

[23] Catala M Kubis N. Gross anatomy and development of the peripheral nervous system. Handb Clin Neurol. (2013) 115:29–41. doi: 10.1016/B978-0-444-52902-2.00003-5 23931773

[24] Kupari J Häring M Agirre E Castelo-Branco G Ernfors P. An atlas of vagal sensory neurons and their molecular specialization. Cell Rep. (2019) 27:2508–23.e4. doi: 10.1016/j.celrep.2019.04.096 31116992 PMC6533201

[25] Saks R Shatzkes D Gillespie J. Pictorial Review of Cranial Nerve Denervation in the Head and Neck. Radiographics. (2024) 44:10. doi: 10.1148/rg.240023 39298352

[26] Vilensky JA. The neglected cranial nerve: nervus terminalis (cranial nerve N). Clin Anat. (2014) 27:46–53. doi: 10.1002/ca.22130 22836597

[27] Su Y Barr J Jaquish A Xu J Verheyden JM Sun X. Identification of lung innervating sensory neurons and their target specificity. Am J Physiol Lung Cell Mol Physiol. (2022) 322:L50–l63. doi: 10.1152/ajplung.00376.2021 34755535 PMC8721910

[28] Phillips RJ Powley TL. Innervation of the gastrointestinal tract: patterns of aging. Auton Neurosci. (2007) 136:1–19. doi: 10.1016/j.autneu.2007.04.005 17537681 PMC2045700

[29] Makhmutova M Caicedo A. Optical imaging of pancreatic innervation. Front Endocrinol (Lausanne). (2021) 12:663022. doi: 10.3389/fendo.2021.663022 33986728 PMC8112238

[30] Browning KN Verheijden S Boeckxstaens GE. The vagus nerve in appetite regulation, mood, and intestinal inflammation. Gastroenterology. (2017) 152:730–44. doi: 10.1053/j.gastro.2016.10.046 PMC5337130 27988382

[31] Paton JF Li YW Kasparov S. Reflex response and convergence of pharyngoesophageal and peripheral chemoreceptors in the nucleus of the solitary tract. Neuroscience. (1999) 93:143–54. doi: 10.1016/S0306-4522(99)00098-6 10430479

[32] Frame AA Carmichael CY Wainford RD. Renal afferents. Curr Hypertens Rep. (2016) 18:69. doi: 10.1007/s11906-016-0676-z 27595156 PMC5011151

[33] Kopp UC. Role of renal sensory nerves in physiological and pathophysiological conditions. Am J Physiol Regul Integr Comp Physiol. (2015) 308:R79–95. doi: 10.1152/ajpregu.00351.2014 PMC4297860 25411364

[34] Garrett A Rakhilin N Wang N McKey J Cofer G Anderson RB. Mapping the peripheral nervous system in the whole mouse via compressed sensing tractography. J Neural Eng. (2021) 18:13. doi: 10.1088/1741-2552/ac0089 PMC12490744 33979784

[35] Wu M Song G Li J Song Z Zhao B Liang L. Innervation of nociceptor neurons in the spleen promotes germinal center responses and humoral immunity. Cell. (2024) 187:2935–51.e19. doi: 10.1016/j.cell.2024.04.027 38772371

[36] Huang S Ziegler CGK Austin J Mannoun N Vukovic M Ordovas-Montanes J. Lymph nodes are innervated by a unique population of sensory neurons with immunomodulatory potential. Cell. (2021) 184:441–59.e25. doi: 10.1016/j.cell.2020.11.028 33333021 PMC9612289

[37] Mazzone SB Undem BJ. Vagal afferent innervation of the airways in health and disease. Physiol Rev. (2016) 96:975–1024. doi: 10.1152/physrev.00039.2015 27279650 PMC4982036

[38] Estivill-Torrús G Martínez-Padilla AB Sánchez-Salido L Evercooren AB García-Díaz B. The dorsal root ganglion as a target for neurorestoration in neuropathic pain. Neural Regener Res. (2024) 19:296–301. doi: 10.4103/1673-5374.374655 PMC10503598 37488881

[39] Young RL Page AJ Cooper NJ Frisby CL Blackshaw LA. Sensory and motor innervation of the crural diaphragm by the vagus nerves. Gastroenterology. (2010) 138:1091–101.e1-5. doi: 10.1053/j.gastro.2009.08.053 19732773

[40] Chen C Sun L Adler A Zhou H Zhang L Zhang L. Synchronized activity of sensory neurons initiates cortical synchrony in a model of neuropathic pain. Nat Commun. (2023) 14:689. doi: 10.1038/s41467-023-36093-z 36755026 PMC9908980

[41] Kollarik M Ru F Brozmanova M. Vagal afferent nerves with the properties of nociceptors. Auton Neurosci. (2010) 153:12–20. doi: 10.1016/j.autneu.2009.08.001 19751993 PMC2818152

[42] Bai L Mesgarzadeh S Ramesh KS Huey EL Liu Y Gray LA. Genetic identification of vagal sensory neurons that control feeding. Cell. (2019) 179:1129–43.e23. doi: 10.1016/j.cell.2019.10.031 31730854 PMC6916730

[43] Verri WA Jr Cunha TM Parada CA Poole S Cunha FQ Ferreira SH. Hypernociceptive role of cytokines and chemokines: targets for analgesic drug development? Pharmacol Ther. (2006) 112:116–38. doi: 10.1016/j.pharmthera.2006.04.001 16730375

[44] Chiu IM von Hehn CA Woolf CJ. Neurogenic inflammation and the peripheral nervous system in host defense and immunopathology. Nat Neurosci. (2012) 15:1063–7. doi: 10.1038/nn.3144 PMC3520068 22837035

[45] Grace PM Hutchinson MR Maier SF Watkins LR. Pathological pain and the neuroimmune interface. Nat Rev Immunol. (2014) 14:217–31. doi: 10.1038/nri3621 PMC5525062 24577438

[46] McMahon SB La Russa F Bennett DL. Crosstalk between the nociceptive and immune systems in host defence and disease. Nat Rev Neurosci. (2015) 16:389–402. doi: 10.1038/nrn3946 26087680

[47] Chavan SS Pavlov VA Tracey KJ. Mechanisms and therapeutic relevance of neuro-immune communication. Immunity. (2017) 46:927–42. doi: 10.1016/j.immuni.2017.06.008 PMC5578398 28636960

[48] Pinho-Ribeiro FA Verri WA Jr. Chiu IM. Nociceptor sensory neuron-immune interactions in pain and inflammation. Trends Immunol. (2017) 38:5–19. doi: 10.1016/j.it.2016.10.001 27793571 PMC5205568

[49] Zhang W Lyu M Bessman NJ Xie Z Arifuzzaman M Yano H. Gut-innervating nociceptors regulate the intestinal microbiota to promote tissue protection. Cell. (2022) 185:4170–89.e20. doi: 10.1016/j.cell.2022.09.008 36240781 PMC9617796

[50] Procaccini C Pucino V De Rosa V Marone G Matarese G. Neuro-endocrine networks controlling immune system in health and disease. Front Immunol. (2014) 5:143. doi: 10.3389/fimmu.2014.00143 24778633 PMC3985001

[51] Belvisi MG. Overview of the innervation of the lung. Curr Opin Pharmacol. (2002) 2:211–5. doi: 10.1016/S1471-4892(02)00145-5 12020459

[52] Brierley SM Jones RC 3rd Gebhart GF Blackshaw LA. Splanchnic and pelvic mechanosensory afferents signal different qualities of colonic stimuli in mice. Gastroenterology. (2004) 127:166–78. doi: 10.1053/j.gastro.2004.04.008 15236183

[53] Oaklander AL Siegel SM. Cutaneous innervation: form and function. J Am Acad Dermatol. (2005) 53:1027–37. doi: 10.1016/j.jaad.2005.08.049 16310064

[54] Zhang FC Weng RX Li D Li YC Dai XX Hu S. A vagus nerve dominant tetra-synaptic ascending pathway for gastric pain processing. Nat Commun. (2024) 15:9824. doi: 10.1038/s41467-024-54056-w 39537596 PMC11561356

[55] Gao X Zhang D Xu C Li H Caron KM Frenette PS. Nociceptive nerves regulate haematopoietic stem cell mobilization. Nature. (2021) 589:591–6. doi: 10.1038/s41586-020-03057-y PMC7856173 33361809

[56] Hoeffel G Debroas G Roger A Rossignol R Gouilly J Laprie C. Sensory neuron-derived TAFA4 promotes macrophage tissue repair functions. Nature. (2021) 594:94–9. doi: 10.1038/s41586-021-03563-7 34012116

[57] Chiu IM Heesters BA Ghasemlou N Von Hehn CA Zhao F Tran J. Bacteria activate sensory neurons that modulate pain and inflammation. Nature. (2013) 501:52–7. doi: 10.1038/nature12479 PMC3773968 23965627

[58] Pinho-Ribeiro FA Baddal B Haarsma R O’Seaghdha M Yang NJ Blake KJ. Blocking neuronal signaling to immune cells treats streptococcal invasive infection. Cell. (2018) 173:1083–97.e22. doi: 10.1016/j.cell.2018.04.006 29754819 PMC5959783

[59] Cohen JA Edwards TN Liu AW Hirai T Jones MR Wu J. Cutaneous TRPV1(+) neurons trigger protective innate type 17 anticipatory immunity. Cell. (2019) 178:919–32.e14. doi: 10.1016/j.cell.2019.06.022 31353219 PMC6788801

[60] Zhang S Edwards TN Chaudhri VK Wu J Cohen JA Hirai T. Nonpeptidergic neurons suppress mast cells via glutamate to maintain skin homeostasis. Cell. (2021) 184:2151–66.e16. doi: 10.1016/j.cell.2021.03.002 33765440 PMC8052305

[61] Tamari M Del Bel KL Ver Heul AM Zamidar L Orimo K Hoshi M. Sensory neurons promote immune homeostasis in the lung. Cell. (2024) 187:44–61.e17. doi: 10.1016/j.cell.2023.11.027 38134932 PMC10811756

[62] Cardoso V Chesné J Ribeiro H García-Cassani B Carvalho T Bouchery T. Neuronal regulation of type 2 innate lymphoid cells via neuromedin U. Nature. (2017) 549:277–81. doi: 10.1038/nature23469 PMC5714273 28869974

[63] Baral P Umans BD Li L Wallrapp A Bist M Kirschbaum T. Nociceptor sensory neurons suppress neutrophil and γδ T cell responses in bacterial lung infections and lethal pneumonia. Nat Med. (2018) 24:417–26. doi: 10.1038/nm.4501 PMC6263165 29505031

[64] Zhu Y Meerschaert KA Galvan-Pena S Bin NR Yang D Basu H. A chemogenetic screen reveals that Trpv1-expressing neurons control regulatory T cells in the gut. Science. (2024) 385:eadk1679. doi: 10.1126/science.adk1679 39088603 PMC11416019

[65] Godinho-Silva C Cardoso F Veiga-Fernandes H. Neuro-immune cell units: A new paradigm in physiology. Annu Rev Immunol. (2019) 37:19–46. doi: 10.1146/annurev-immunol-042718-041812 30379595

[66] Veiga-Fernandes H Pachnis V. Neuroimmune regulation during intestinal development and homeostasis. Nat Immunol. (2017) 18:116–22. doi: 10.1038/ni.3634 28092371

[67] Hanč P Gonzalez RJ Mazo IB Wang Y Lambert T Ortiz G. Multimodal control of dendritic cell functions by nociceptors. Science. (2023) 379:eabm5658. doi: 10.1126/science.abm5658 36996219 PMC10642951

[68] Eyerich S Eyerich K Traidl-Hoffmann C Biedermann T. Cutaneous barriers and skin immunity: differentiating A connected network. Trends Immunol. (2018) 39:315–27. doi: 10.1016/j.it.2018.02.004 29551468

[69] Ishikawa H Barber GN. STING is an endoplasmic reticulum adaptor that facilitates innate immune signalling. Nature. (2008) 455:674–8. doi: 10.1038/nature07317 PMC2804933 18724357

[70] Woo SR Fuertes MB Corrales L Spranger S Furdyna MJ Leung MY. STING-dependent cytosolic DNA sensing mediates innate immune recognition of immunogenic tumors. Immunity. (2014) 41:830–42. doi: 10.1016/j.immuni.2014.10.017 PMC4384884 25517615

[71] Donnelly CR Jiang C Andriessen AS Wang K Wang Z Ding H. STING controls nociception via type I interferon signalling in sensory neurons. Nature. (2021) 591:275–80. doi: 10.1038/s41586-020-03151-1 PMC7977781 33442058

[72] Warr MR Pietras EM Passegué E. Mechanisms controlling hematopoietic stem cell functions during normal hematopoiesis and hematological Malignancies. Wiley Interdiscip Rev Syst Biol Med. (2011) 3:681–701. doi: 10.1002/wsbm.v3.6 21412991

[73] Katayama Y Battista M Kao WM Hidalgo A Peired AJ Thomas SA. Signals from the sympathetic nervous system regulate hematopoietic stem cell egress from bone marrow. Cell. (2006) 124:407–21. doi: 10.1016/j.cell.2005.10.041 16439213

[74] Lucas D Scheiermann C Chow A Kunisaki Y Bruns I Barrick C. Chemotherapy-induced bone marrow nerve injury impairs hematopoietic regeneration. Nat Med. (2013) 19:695–703. doi: 10.1038/nm.3155 23644514 PMC3964478

[75] Maryanovich M Zahalka AH Pierce H Pinho S Nakahara F Asada N. Adrenergic nerve degeneration in bone marrow drives aging of the hematopoietic stem cell niche. Nat Med. (2018) 24:782–91. doi: 10.1038/s41591-018-0030-x PMC6095812 29736022

[76] Bassi GS Kanashiro A Coimbra NC Terrando N Maixner W Ulloa L. Anatomical and clinical implications of vagal modulation of the spleen. Neurosci Biobehav Rev. (2020) 112:363–73. doi: 10.1016/j.neubiorev.2020.02.011 PMC7211143 32061636

[77] Ulloa L. Bioelectronic neuro-immunology: Neuronal networks for sympathetic-splenic and vagal-adrenal control. Neuron. (2023) 111:10–4. doi: 10.1016/j.neuron.2022.09.015 36202096

[78] Liu S Wang ZF Su YS Ray RS Jing XH Wang YQ. Somatotopic organization and intensity dependence in driving distinct NPY-expressing sympathetic pathways by electroacupuncture. Neuron. (2020) 108:436–50.e7. doi: 10.1016/j.neuron.2020.07.015 32791039 PMC7666081

[79] Zhang X Lei B Yuan Y Zhang L Hu L Jin S. Brain control of humoral immune responses amenable to behavioural modulation. Nature. (2020) 581:204–8. doi: 10.1038/s41586-020-2235-7 32405000

[80] Pavlov VA Chavan SS Tracey KJ. Molecular and functional neuroscience in immunity. Annu Rev Immunol. (2018) 36:783–812. doi: 10.1146/annurev-immunol-042617-053158 29677475 PMC6057146

[81] Felten DL Felten SY Bellinger DL Lorton D. Noradrenergic and peptidergic innervation of secondary lymphoid organs: role in experimental rheumatoid arthritis. Eur J Clin Invest. (1992) 22 Suppl 1:37–41. 1281104

[82] Fink T Weihe E. Multiple neuropeptides in nerves supplying mammalian lymph nodes: messenger candidates for sensory and autonomic neuroimmunomodulation? Neurosci Lett. (1988) 90:39–44. doi: 10.1016/0304-3940(88)90783-5 2457855

[83] Lorton D Lubahn C Engan C Schaller J Felten DL Bellinger DL. Local application of capsaicin into the draining lymph nodes attenuates expression of adjuvant-induced arthritis. Neuroimmunomodulation. (2000) 7:115–25. doi: 10.1159/000026429 10754399

[84] Hanes WM Olofsson PS Talbot S Tsaava T Ochani M Imperato GH. Neuronal circuits modulate antigen flow through lymph nodes. Bioelectron Med. (2016) 3:18–28. doi: 10.15424/bioelectronmed.2016.00001 33145374 PMC7604943

[85] Munger BL Ide C. The structure and function of cutaneous sensory receptors. Arch Histol Cytol. (1988) 51:1–34. doi: 10.1679/aohc.51.1 3137944

[86] Kelly EJ Terenghi G Hazari A Wiberg M. Nerve fibre and sensory end organ density in the epidermis and papillary dermis of the human hand. Br J Plast Surg. (2005) 58:774–9. doi: 10.1016/j.bjps.2004.12.017 16086989

[87] Paus R Theoharides TC Arck PC. Neuroimmunoendocrine circuitry of the ‘brain-skin connection’. Trends Immunol. (2006) 27:32–9. doi: 10.1016/j.it.2005.10.002 16269267

[88] Winkelmann RK. Cutaneous sensory nerves. Semin Dermatol. (1988) 7:236–68. 3153451

[89] Kaewpitak A Bauer CS Seward EP Boissonade FM Douglas CWI. Porphyromonas gingivalis lipopolysaccharide rapidly activates trigeminal sensory neurons and may contribute to pulpal pain. Int Endod J. (2020) 53:846–58. doi: 10.1111/iej.13282 32058593

[90] Ohara K Shimizu K Matsuura S Ogiso B Omagari D Asano M. Toll-like receptor 4 signaling in trigeminal ganglion neurons contributes tongue-referred pain associated with tooth pulp inflammation. J Neuroinflammation. (2013) 10:139. doi: 10.1186/1742-2094-10-139 24267924 PMC4222866

[91] Diogenes A Ferraz CC Akopian AN Henry MA Hargreaves KM. LPS sensitizes TRPV1 via activation of TLR4 in trigeminal sensory neurons. J Dent Res. (2011) 90:759–64. doi: 10.1177/0022034511400225 21393555

[92] Meseguer V Alpizar YA Luis E Tajada S Denlinger B Fajardo O. TRPA1 channels mediate acute neurogenic inflammation and pain produced by bacterial endotoxins. Nat Commun. (2014) 5:3125. doi: 10.1038/ncomms4125 24445575 PMC3905718

[93] West PW Canning BJ Merlo-Pich E Woodcock AA Smith JA. Morphologic characterization of nerves in whole-mount airway biopsies. Am J Respir Crit Care Med. (2015) 192:30–9. doi: 10.1164/rccm.201412-2293OC PMC4511424 25906337

[94] Dinh QT Groneberg DA Peiser C Mingomataj E Joachim RA Witt C. Substance P expression in TRPV1 and trkA-positive dorsal root ganglion neurons innervating the mouse lung. Respir Physiol Neurobiol. (2004) 144:15–24. doi: 10.1016/j.resp.2004.08.001 15522699

[95] Chang RB Strochlic DE Williams EK Umans BD Liberles SD. Vagal sensory neuron subtypes that differentially control breathing. Cell. (2015) 161:622–33. doi: 10.1016/j.cell.2015.03.022 PMC4842319 25892222

[96] Coleridge HM Coleridge JC Roberts AM. Rapid shallow breathing evoked by selective stimulation of airway C fibres in dogs. J Physiol. (1983) 340:415–33. doi: 10.1113/jphysiol.1983.sp014770 PMC1199217 6887055

[97] Canning BJ Mori N Mazzone SB. Vagal afferent nerves regulating the cough reflex. Respir Physiol Neurobiol. (2006) 152:223–42. doi: 10.1016/j.resp.2006.03.001 16740418

[98] Widdicombe JH Wine JJ. Airway gland structure and function. Physiol Rev. (2015) 95:1241–319. doi: 10.1152/physrev.00039.2014 26336032

[99] Drake MG Scott GD Blum ED Lebold KM Nie Z Lee JJ. Eosinophils increase airway sensory nerve density in mice and in human asthma. Sci Transl Med. (2018) 10:457. doi: 10.1126/scitranslmed.aar8477 PMC6592848 30185653

[100] Wallrapp A Burkett PR Riesenfeld SJ Kim SJ Christian E Abdulnour RE. Calcitonin gene-related peptide negatively regulates alarmin-driven type 2 innate lymphoid cell responses. Immunity. (2019) 51:709–23.e6. doi: 10.1016/j.immuni.2019.09.005 31604686 PMC7076585

[101] Nagashima H Mahlakõiv T Shih HY Davis FP Meylan F Huang Y. Neuropeptide CGRP limits group 2 innate lymphoid cell responses and constrains type 2 inflammation. Immunity. (2019) 51:682–95.e6. doi: 10.1016/j.immuni.2019.06.009 31353223 PMC6801073

[102] Jung WJ Lee SY Choi SI Kim BK Lee EJ In KH. Toll-like receptor expression in pulmonary sensory neurons in the bleomycin-induced fibrosis model. PLoS One. (2018) 13:e0193117. doi: 10.1371/journal.pone.0193117 29518161 PMC5843166

[103] Duan JX Zhou Y Zhou AY Guan XX Liu T Yang HH. Calcitonin gene-related peptide exerts anti-inflammatory property through regulating murine macrophages polarization *in vitro* . Mol Immunol. (2017) 91:105–13. doi: 10.1016/j.molimm.2017.08.020 28892747

[104] Zhu F Yu D Qin X Qian Y Ma J Li W. The neuropeptide CGRP enters the macrophage cytosol to suppress the NLRP3 inflammasome during pulmonary infection. Cell Mol Immunol. (2023) 20:264–76. doi: 10.1038/s41423-022-00968-w PMC9970963 36600053

[105] Bin NR Prescott SL Horio N Wang Y Chiu IM Liberles SD. An airway-to-brain sensory pathway mediates influenza-induced sickness. Nature. (2023) 615:660–7. doi: 10.1038/s41586-023-05796-0 PMC10033449 36890237

[106] Su Y Xu J Zhu Z Chin J Xu L Yu H. Brainstem Dbh(+) neurons control allergen-induced airway hyperreactivity. Nature. (2024) 631:601–9. doi: 10.1038/s41586-024-07608-5 PMC11254774 38987587

[107] Sharkey KA Mawe GM. The enteric nervous system. Physiol Rev. (2023) 103:1487–564. doi: 10.1152/physrev.00018.2022 PMC9970663 36521049

[108] Robinson DR McNaughton PA Evans ML Hicks GA. Characterization of the primary spinal afferent innervation of the mouse colon using retrograde labelling. Neurogastroenterol Motil. (2004) 16:113–24. doi: 10.1046/j.1365-2982.2003.00456.x 14764211

[109] Spencer NJ Hu H. Enteric nervous system: sensory transduction, neural circuits and gastrointestinal motility. Nat Rev Gastroenterol Hepatol. (2020) 17:338–51. doi: 10.1038/s41575-020-0271-2 PMC7474470 32152479

[110] Wolfson RL Abdelaziz A Rankin G Kushner S Qi L Mazor O. DRG afferents that mediate physiologic and pathologic mechanosensation from the distal colon. Cell. (2023) 186:3368–85.e18. doi: 10.1016/j.cell.2023.07.007 37541195 PMC10440726

[111] Ranade SS Woo SH Dubin AE Moshourab RA Wetzel C Petrus M. Piezo2 is the major transducer of mechanical forces for touch sensation in mice. Nature. (2014) 516:121–5. doi: 10.1038/nature13980 PMC4380172 25471886

[112] Xie Z Feng J Hibberd TJ Chen BN Zhao Y Zang K. Piezo2 channels expressed by colon-innervating TRPV1-lineage neurons mediate visceral mechanical hypersensitivity. Neuron. (2023) 111:526–38.e4. doi: 10.1016/j.neuron.2022.11.015 36563677 PMC9957938

[113] Wang P Jia Y Liu T Jan YN Zhang W. Visceral mechano-sensing neurons control drosophila feeding by using Piezo as a sensor. Neuron. (2020) 108:640–50.e4. doi: 10.1016/j.neuron.2020.08.017 32910893 PMC8386590

[114] Servin-Vences MR Lam RM Koolen A Wang Y Saade DN Loud M. PIEZO2 in somatosensory neurons controls gastrointestinal transit. Cell. (2023) 186:3386–99.e15. doi: 10.1016/j.cell.2023.07.006 37541196 PMC10501318

[115] Chu C Artis D Chiu IM. Neuro-immune interactions in the tissues. Immunity. (2020) 52:464–74. doi: 10.1016/j.immuni.2020.02.017 PMC10710744 32187517

[116] Mashaghi A Marmalidou A Tehrani M Grace PM Pothoulakis C Dana R. Neuropeptide substance P and the immune response. Cell Mol Life Sci. (2016) 73:4249–64. doi: 10.1007/s00018-016-2293-z PMC5056132 27314883

[117] Choi JE Di Nardo A. Skin neurogenic inflammation. Semin Immunopathol. (2018) 40:249–59. doi: 10.1007/s00281-018-0675-z PMC6047518 29713744

[118] Forster PM Jakob MO Yusuf D Bubeck M Limberger H Luo Y. A transcriptional atlas of gut-innervating neurons reveals activation of interferon signaling and ferroptosis during intestinal inflammation. Neuron. (2025). doi: 10.1016/j.neuron.2025.02.018 40101721

[119] Teratani T Mikami Y Nakamoto N Suzuki T Harada Y Okabayashi K. The liver-brain-gut neural arc maintains the T(reg) cell niche in the gut. Nature. (2020) 585:591–6. doi: 10.1038/s41586-020-2425-3 32526765

[120] Xie Z Zhang X Zhao M Huo L Huang M Li D. The gut-to-brain axis for toxin-induced defensive responses. Cell. (2022) 185:4298–316.e21. doi: 10.1016/j.cell.2022.10.001 36323317

[121] Ichiki T Wang T Kennedy A Pool AH Ebisu H Anderson DJ. Sensory representation and detection mechanisms of gut osmolality change. Nature. (2022) 602:468–74. doi: 10.1038/s41586-021-04359-5 35082448

[122] Rawat A Morrison BM. Metabolic transporters in the peripheral nerve-what, where, and why? Neurotherapeutics. (2021) 18:2185–99. doi: 10.1007/s13311-021-01150-2 PMC8804006 34773210

[123] Myers MG Jr. Olson DP. Central nervous system control of metabolism. Nature. (2012) 491:357–63. doi: 10.1038/nature11705 23151578

[124] Xu Y Elmquist JK Fukuda M. Central nervous control of energy and glucose balance: focus on the central melanocortin system. Ann N Y Acad Sci. (2011) 1243:1–14. doi: 10.1111/j.1749-6632.2011.06248.x 22211889 PMC3467098

[125] Guilherme A Henriques F Bedard AH Czech MP. Molecular pathways linking adipose innervation to insulin action in obesity and diabetes mellitus. Nat Rev Endocrinol. (2019) 15:207–25. doi: 10.1038/s41574-019-0165-y 30733616 PMC7073451

[126] Cheng W Gordian D Ludwig MQ Pers TH Seeley RJ Myers MG Jr. Hindbrain circuits in the control of eating behaviour and energy balance. Nat Metab. (2022) 4:826–35. doi: 10.1038/s42255-022-00606-9 35879458

[127] Nguyen NLT Xue B Bartness TJ. Sensory denervation of inguinal white fat modifies sympathetic outflow to white and brown fat in Siberian hamsters. Physiol Behav. (2018) 190:28–33. doi: 10.1016/j.physbeh.2018.02.019 29447836 PMC5924716

[128] Vaughan CH Bartness TJ. Anterograde transneuronal viral tract tracing reveals central sensory circuits from brown fat and sensory denervation alters its thermogenic responses. Am J Physiol Regul Integr Comp Physiol. (2012) 302:R1049–58. doi: 10.1152/ajpregu.00640.2011 PMC3362143 22378771

[129] Shi M Huang XY Ren XY Wei XY Ma Y Lin ZZ. AIDA directly connects sympathetic innervation to adaptive thermogenesis by UCP1. Nat Cell Biol. (2021) 23:268–77. doi: 10.1038/s41556-021-00642-9 33664495

[130] Himms-Hagen J Cui J Lynn Sigurdson S. Sympathetic and sensory nerves in control of growth of brown adipose tissue: Effects of denervation and of capsaicin. Neurochem Int. (1990) 17:271–9. doi: 10.1016/0197-0186(90)90149-N 20504627

[131] Osaka T Kobayashi A Namba Y Ezaki O Inoue S Kimura S. Temperature- and capsaicin-sensitive nerve fibers in brown adipose tissue attenuate thermogenesis in the rat. Pflugers Arch. (1998) 437:36–42. doi: 10.1007/s004240050743 9817783

[132] Mishra G Townsend KL. The metabolic and functional roles of sensory nerves in adipose tissues. Nat Metab. (2023) 5:1461–74. doi: 10.1038/s42255-023-00868-x PMC12636859 37709960

[133] Foster MT Bartness TJ. Sympathetic but not sensory denervation stimulates white adipocyte proliferation. Am J Physiol Regul Integr Comp Physiol. (2006) 291:R1630–7. doi: 10.1152/ajpregu.00197.2006 16887921

[134] Wang Y Leung VH Zhang Y Nudell VS Loud M Servin-Vences MR. The role of somatosensory innervation of adipose tissues. Nature. (2022) 609:569–74. doi: 10.1038/s41586-022-05137-7 PMC9477745 36045288

[135] Borgmann D Ciglieri E Biglari N Brandt C Cremer AL Backes H. Gut-brain communication by distinct sensory neurons differently controls feeding and glucose metabolism. Cell Metab. (2021) 33:1466–82.e7. doi: 10.1016/j.cmet.2021.05.002 34043943 PMC8280952

[136] Ansari S Khoo B Tan T. Targeting the incretin system in obesity and type 2 diabetes mellitus. Nat Rev Endocrinol. (2024) 20:447–59. doi: 10.1038/s41574-024-00979-9 38632474

[137] Romaní-Pérez M Bullich-Vilarrubias C López-Almela I Sanz Y. The ablation of sensory neurons expressing the nav1.8 sodium channel improves glucose homeostasis and amplifies the GLP-1 signaling in obese female mice. Mol Nutr Food Res. (2024) 68:e2300474. doi: 10.1002/mnfr.202300474 38038153

[138] Magnon C Hall SJ Lin J Xue X Gerber L Freedland SJ. Autonomic nerve development contributes to prostate cancer progression. Science. (2013) 341:1236361. doi: 10.1126/science.1236361 23846904

[139] Ayala GE Dai H Powell M Li R Ding Y Wheeler TM. Cancer-related axonogenesis and neurogenesis in prostate cancer. Clin Cancer Res. (2008) 14:7593–603. doi: 10.1158/1078-0432.CCR-08-1164 19047084

[140] Mauffrey P Tchitchek N Barroca V Bemelmans AP Firlej V Allory Y. Progenitors from the central nervous system drive neurogenesis in cancer. Nature. (2019) 569:672–8. doi: 10.1038/s41586-019-1219-y 31092925

[141] Balood M Ahmadi M Eichwald T Ahmadi A Majdoubi A Roversi K. Nociceptor neurons affect cancer immunosurveillance. Nature. (2022) 611:405–12. doi: 10.1038/s41586-022-05374-w PMC9646485 36323780

[142] Amit M Takahashi H Dragomir MP Lindemann A Gleber-Netto FO Pickering CR. Loss of p53 drives neuron reprogramming in head and neck cancer. Nature. (2020) 578:449–54. doi: 10.1038/s41586-020-1996-3 PMC9723538 32051587

[143] Zhi X Wu F Qian J Ochiai Y Lian G Malagola E. Nociceptive neurons promote gastric tumour progression via a CGRP-RAMP1 axis. Nature. (2025) 640:802–10. doi: 10.1038/s41586-025-08591-1 PMC13022952 39972142

[144] Wang K Ni B Xie Y Li Z Yuan L Meng C. Nociceptor neurons promote PDAC progression and cancer pain by interaction with cancer-associated fibroblasts and suppression of natural killer cells. Cell Res. (2025) 35:362–80. doi: 10.1038/s41422-025-01098-4 PMC12012126 40122998

[145] Padmanaban V Keller I Seltzer ES Ostendorf BN Kerner Z Tavazoie SF. Neuronal substance P drives metastasis through an extracellular RNA-TLR7 axis. Nature. (2024) 633:207–15. doi: 10.1038/s41586-024-07767-5 PMC11633843 39112700

[146] Aalkjær C Nilsson H De Mey JGR. Sympathetic and sensory-motor nerves in peripheral small arteries. Physiol Rev. (2021) 101:495–544. doi: 10.1152/physrev.00007.2020 33270533

[147] Leroux A Paiva Dos Santos B Leng J Oliveira H Amédée J. Sensory neurons from dorsal root ganglia regulate endothelial cell function in extracellular matrix remodelling. Cell Commun Signal. (2020) 18:162. doi: 10.1186/s12964-020-00656-0 33076927 PMC7574530

[148] Zhu S Zidan A Pang K Musayeva A Kang Q Yin J. Promotion of corneal angiogenesis by sensory neuron-derived calcitonin gene-related peptide. Exp Eye Res. (2022) 220:109125. doi: 10.1016/j.exer.2022.109125 35618042 PMC9428938

[149] Brain SD Williams TJ Tippins JR Morris HR MacIntyre I. Calcitonin gene-related peptide is a potent vasodilator. Nature. (1985) 313:54–6. doi: 10.1038/313054a0 3917554

[150] Nuki Y Kawasaki H Taguchi T Takasaki K Wada A. Effects of dorsal rhizotomy on depressor response to spinal cord stimulation mediated by endogenous calcitonin gene-related peptide in the pithed rat. J Neurosurg. (1993) 79:899–904. doi: 10.3171/jns.1993.79.6.0899 7902428

[151] Kawasaki H Nuki C Saito A Takasaki K. Role of calcitonin gene-related peptide-containing nerves in the vascular adrenergic neurotransmission. J Pharmacol Exp Ther. (1990) 252:403–9. doi: 10.1016/S0022-3565(25)13362-4 1688944

[152] Takenaga M Kawasaki H. Endogenous calcitonin gene-related peptide suppresses vasoconstriction mediated by adrenergic nerves in rat mesenteric resistance blood vessels. Eur J Pharmacol. (1999) 367:239–45. doi: 10.1016/S0014-2999(98)00949-2 10078998

[153] Morelli C Castaldi L Brown SJ Streich LL Websdale A Taberner FJ. Identification of a population of peripheral sensory neurons that regulates blood pressure. Cell Rep. (2021) 35:109191. doi: 10.1016/j.celrep.2021.109191 34077727 PMC8187988

[154] Liu L Dana R Yin J. Sensory neurons directly promote angiogenesis in response to inflammation via substance P signaling. FASEB J. (2020) 34:6229–43. doi: 10.1096/fj.201903236R PMC7200295 32162744

[155] Lovelace JW Ma J Yadav S Chhabria K Shen H Pang Z. Vagal sensory neurons mediate the Bezold-Jarisch reflex and induce syncope. Nature. (2023) 623:387–96. doi: 10.1038/s41586-023-06680-7 PMC10632149 37914931

[156] Mark AL. The Bezold-Jarisch reflex revisited: clinical implications of inhibitory reflexes originating in the heart. J Am Coll Cardiol. (1983) 1:90–102. doi: 10.1016/S0735-1097(83)80014-X 6826948

[157] Bonaz B Lane RD Oshinsky ML Kenny PJ Sinha R Mayer EA. Diseases, disorders, and comorbidities of interoception. Trends Neurosci. (2021) 44:39–51. doi: 10.1016/j.tins.2020.09.009 33378656

[158] Randhawa PK Jaggi AS. TRPV1 and TRPV4 channels: potential therapeutic targets for ischemic conditioning-induced cardioprotection. Eur J Pharmacol. (2015) 746:180–5. doi: 10.1016/j.ejphar.2014.11.010 25449039

[159] Maggi CA Meli A. The sensory-efferent function of capsaicin-sensitive sensory neurons. Gen Pharmacol. (1988) 19:1–43. doi: 10.1016/0306-3623(88)90002-X 3278943

[160] Holzer P. Local effector functions of capsaicin-sensitive sensory nerve endings: involvement of tachykinins, calcitonin gene-related peptide and other neuropeptides. Neuroscience. (1988) 24:739–68. doi: 10.1016/0306-4522(88)90064-4 3288903

[161] Varró A Lathrop DA Hester SB Nánási PP Papp JG. Ionic currents and action potentials in rabbit, rat, and Guinea pig ventricular myocytes. Basic Res Cardiol. (1993) 88:93–102. doi: 10.1007/BF00798257 8389123

[162] Mohanta SK Peng L Li Y Lu S Sun T Carnevale L. Neuroimmune cardiovascular interfaces control atherosclerosis. Nature. (2022) 605:152–9. doi: 10.1038/s41586-022-04673-6 35477759

[163] Wang Y Cui L Xu H Liu S Zhu F Yan F. TRPV1 agonism inhibits endothelial cell inflammation via activation of eNOS/NO pathway. Atherosclerosis. (2017) 260:13–9. doi: 10.1016/j.atherosclerosis.2017.03.016 28324760

[164] Chen KS Chen PN Hsieh YS Lin CY Lee YH Chu SC. Capsaicin protects endothelial cells and macrophage against oxidized low-density lipoprotein-induced injury by direct antioxidant action. Chem Biol Interact. (2015) 228:35–45. doi: 10.1016/j.cbi.2015.01.007 25603234

[165] Sun M Mao S Wu C Zhao X Guo C Hu J. Piezo1-mediated neurogenic inflammatory cascade exacerbates ventricular remodeling after myocardial infarction. Circulation. (2024) 149:1516–33. doi: 10.1161/CIRCULATIONAHA.123.065390 38235590

[166] Huntley MA Lou M Goldstein LD Lawrence M Dijkgraaf GJ Kaminker JS. Complex regulation of ADAR-mediated RNA-editing across tissues. BMC Genomics. (2016) 17:61. doi: 10.1186/s12864-015-2291-9 26768488 PMC4714477

[167] Jo YH. Differential transcriptional profiles of vagal sensory neurons in female and male mice. Front Neurosci. (2024) 18:1393196. doi: 10.3389/fnins.2024.1393196 38808032 PMC11131592

[168] N’Guetta PY McLarnon SR Tassou A Geron M Shirvan S Hill RZ. Comprehensive mapping of sensory and sympathetic innervation of the developing kidney. Cell Rep. (2024) 43:114860. doi: 10.1016/j.celrep.2024.114860 39412983 PMC11616766

[169] Ruhl CR Pasko BL Khan HS Kindt LM Stamm CE Franco LH. Mycobacterium tuberculosis sulfolipid-1 activates nociceptive neurons and induces cough. Cell. (2020) 181:293–305.e11. doi: 10.1016/j.cell.2020.02.026 32142653 PMC7102531

[170] Granton E Brown L Defaye M Moazen P Almblad H Randall TE. Biofilm exopolysaccharides alter sensory-neuron-mediated sickness during lung infection. Cell. (2024) 187:1874–88.e14. doi: 10.1016/j.cell.2024.03.001 38518773

[171] Muller PA Schneeberger M Matheis F Wang P Kerner Z Ilanges A. Microbiota modulate sympathetic neurons via a gut-brain circuit. Nature. (2020) 583:441–6. doi: 10.1038/s41586-020-2474-7 PMC7367767 32641826

